# Phytochemical Composition, Antioxidant Capacity, and Enzyme Inhibitory Activity in Callus, Somaclonal Variant, and Normal Green Shoot Tissues of *Catharanthus roseus* (L) G. Don

**DOI:** 10.3390/molecules25214945

**Published:** 2020-10-26

**Authors:** O. New Lee, Gunes Ak, Gokhan Zengin, Zoltán Cziáky, József Jekő, Kannan R.R. Rengasamy, Han Yong Park, Doo Hwan Kim, Iyyakkannu Sivanesan

**Affiliations:** 1 Department of Bioindustry and Bioresource Engineering, Sejong University, 209 Neungdong-ro, Gwangjin-gu, Seoul 05006, Korea; onewlee@sejong.ac.kr (O.N.L.); hypark@sejong.ac.kr (H.Y.P.); 2 Department of Biology, Faculty of Science, Selcuk University, Konya 42130, Turkey; akguneselcuk@gmail.com (G.A.); gokhanzengin@selcuk.edu.tr (G.Z.); 3 Agricultural and Molecular Research and Service Institute, University of Nyíregyháza, 4400 Nyíregyháza, Hungary; cziaky.zoltan@nye.hu (Z.C.); jjozsi@gmail.com (J.J.); 4 Indigenous Knowledge Systems Centre, Faculty of Natural and Agricultural Sciences, North-West University, Private Bag X2046, Mmabatho 2745, North West, South Africa; Ragupathi.Rengasamy@nwu.ac.za; 5 Department of Bioresources and Food Science, Institute of Natural Science and Agriculture, Konkuk University, Seoul 05029, Korea; kimdh@konkuk.ac.kr

**Keywords:** alkaloids, antioxidant activity, in vitro flowering, micropropagation, phenolics, somaclonal variation

## Abstract

This study aimed to investigate the impact of plant growth regulators, sucrose concentration, and the number of subcultures on axillary shoot multiplication, in vitro flowering, and somaclonal variation and to assess the phytochemical composition, antioxidant capacity, and enzyme inhibitory potential of in vitro-established callus, somaclonal variant, and normal green shoots of *Catharanthus roseus*. The highest shoot induction rate (95.8%) and highest number of shoots (23.6), with a mean length of 4.5 cm, were attained when the *C. roseus* nodal explants (0.6–1 cm in length) were cultivated in Murashige and Skoog (MS) medium with 2 µM thidiazuron, 1 µM 2-(1-naphthyl) acetic acid (NAA), and 4% sucrose. The in vitro flowering of *C. roseus* was affected by sucrose, and the number of subcultures had a significant effect on shoot multiplication and somaclonal variation. The highest levels of phenolics and flavonoids were found in normal green shoots, followed by those in somaclonal variant shoots and callus. The phytochemicals in *C. roseus* extracts were qualified using liquid chromatography–tandem mass spectrometry. A total of 39, 55, and 59 compounds were identified in the callus, somaclonal variant shoot, and normal green shoot tissues, respectively. The normal green shoot extracts exhibited the best free radical scavenging ability and reducing power activity. The strongest acetylcholinesterase inhibitory effects were found in the callus, with an IC50 of 0.65 mg/mL.

Academic editors: Christophe Hano; Bilal Haider Abbasi; Marcello Iriti

## 1. Introduction

*Catharanthus roseus* (L) G. Don (Family: Apocynaceae), also known as periwinkle, is an attractive, evergreen herb. It grows to approximately 100 cm in height and is native to Madagascar. Periwinkle is a source of commercial bioactive alkaloids, including vinblastine and vincristine, which have anti-cancer activities [1,2]. It also contains several important bioactive compounds, such as anthocyanins, flavonol glycosides, phenolic acids, saponins, steroids, and terpenoids, that exhibit antidiarrheal, antidiabetic, antihypoglycemic, antimicrobial, wound healing, and antioxidant activities [3,4,5,6,7,8,9]. *C. roseus* blooms throughout the year with pink, purple, or white fragrant flowers, which have high ornamental value. It is commonly cultivated as an ornamental and medicinal plant in Africa, Australia, China, Europe, and the United States [10]. It is naturally propagated by seeds or cuttings, but a shortage of healthy seeds and cuttings has affected its extensive propagation. Additionally, the large-scale commercial production of new cultivars with medicinal or ornamental value, raised by traditional methods, is time-consuming. Furthermore, the marketable production of *C. roseus* metabolites is often restricted by low levels of medicinal compounds. However, the limitations of conventional propagational methods may be overcome by in vitro culturing. Micropropagation is an effective in vitro technique for the rapid commercial production of plantlets and bioactive metabolites. Several studies have attempted to micropropagate *C. roseus* using plant tissue culture [9,11].

The production of *C. roseus* phytochemicals has been accomplished using callus, cell suspension, somatic embryo, and transformed or non-transformed root and shoot cultures by optimizing the chemical and physical parameters [7,9,12,13]. Several alkaloids, such as ajmalicine, vindoline, catharanthine, vinblastine, and vincristine, were successfully obtained from *C. roseus* shoot cultures [14,15,16,17,18,19,20]. Phenolics are essential secondary metabolites obtained from various plant parts and have a wide range of biological activities [21]. Several phenolic compounds have been obtained in vitro, mostly from callus and cell suspension cultures of *C. roseus* [3,7,9]. However, information on the production of phenolics from *C. roseus* shoot cultures has never been reported, except for the identification of 2,3-dihydroxybenzoic acid from *C. roseus* shoot cultures [22]. To date, the phenolic profile of *C. roseus* shoot cultures has not been documented. Therefore, it is necessary to develop effective analysis procedures for bioactive compounds, including phenolics, in *C. roseus* shoot cultures, for the large-scale commercial production of phytochemicals. The mass production of shoots in vitro often depends on explant type, plant growth regulators (PGRs), sucrose, and the number of subcultures [23].

Explants with vegetative meristems are often suitable for axillary shoot multiplication and clonal propagation. Direct multiple shoot regeneration has been achieved using nodal segments, shoot tips, and axillary buds from *C. roseus* seedlings and mature plants [11,20,24,25,26,27,28]. Cytokinins play an essential role in shoot development. N^6^-benzyladenine (BA) [19,25,26,27], N^6^-furfuryladenine (Kinetin) [19,24,26,27], and thidiazuron (1-phenyl-3-(1,2,3,-thiadiazol-5-yl)urea, TDZ) [19] are used to induce multiple shoots in *C. roseus*. TDZ (substituted phenyl urea) is more efficient at multiple shoot formation in several shrubs, including *C. roseus* [19,29,30]. Moreover, TDZ supplementation increases the phytochemical content of in vitro cultures by altering various physiological activities [30,31]. However, high-dose or continuous TDZ exposure results in growth inhibition, leaf chlorosis, and hyperhydricity in explants containing media [29,32]. Thus, identifying the optimal dose of TDZ is necessary for healthy mass shoot production.

Sucrose is a frequent carbon source in tissue culture media that plays an important role in culture initiation and development and metabolite production [33]. High-level sucrose supplementation (6%) enhances the biomass and phytochemical content of *C. roseus* cell suspension cultures [34,35], while low-dose sucrose supplementation (2%) has been used in woody plant medium for adventitious shoot regeneration in *C. roseus* [36]. Other carbon sources also affect the somatic embryo maturation of *C. roseus* [37]. To the best of our knowledge, the effects of sucrose on axillary shoot proliferation in *C. roseus* have not been reported.

Variations in plant in vitro cultures are called somaclonal variation (SV). SV is a severe problem for the extensive micropropagation of elite genotypes but can also be used in plant improvement programs. The incidence of SV is higher in callus and indirectly regenerated shoots than in axillary shoot cultures. However, the rate of SV in in vitro cultures depends on the plant species, cultivar, culture conditions, growth media components, and the number of subcultures [38,39,40,41]. The effects of subculturing on *C. roseus* shoot multiplication have received little attention and the SV of multiple *C. roseus* shoot cultures is unreported.

Prior studies of the in vitro micropropagation of *C. roseus* have shown that axillary shoot multiplication depends on the explant source, genotype, plant growth regulators, and the components of the culture media. To date, the simultaneous detection of important phytochemicals, such as alkaloids and phenolics, in *C. roseus* callus and shoot cultures has not been documented. The objectives of this study were (1) to evaluate the effects of the plant growth regulators, the sucrose concentration, and the number of subcultures on in vitro micropropagation, (2) to document SV in axillary shoot cultures, (3) to assess the phytochemical composition of in vitro-established callus, somaclonal variant, and normal green shoots, and (4) to evaluate the antioxidant capacity and enzyme inhibitory potential of *C. roseus*.

## 2. Results

### 2.1. In Vitro Micropropagation

The surface disinfection technique produced 91% germ-free explants. Nodal explants of *C. roseus* were cultivated on Murashige and Skoog (MS) medium containing 0–16 µM of cytokinin for axillary shoot multiplication. Shoot initiation was observed within 14 days of cultivation. The cytokinins, their concentration, and the interactions significantly (*p* ≤ 0.001) affected the induction and development of axillary shoots (Table 1). The presence of 1–16 µM BA in the medium improved the axillary shoot multiplication compared to control (devoid of BA). The rate of shoot initiation (66.4%) and the number of shoots (6.3) in the MS medium with 4 µM BA were higher than the other BA treatments. The longest shoot length (3.1 cm) was attained on basal medium with 2 µM BA (Table 1).

The addition of 1–16 µM kinetin also promotes multiple shoot production in *C. roseus*, the rate of shoot induction ranged from 25.7% to 59.5%, and the number of shoots produced ranged from 2.3 to 5.7, with an average length of 1.5–3.6 cm (Table 1). The inclusion of 1–16 µM TDZ in the medium increased the percentage of multiple shoot regeneration compared to the control (without TDZ). The shoot induction rate (75.2%), the number of shoots (10.1), and shoot elongation (5.0 cm) in the MS medium with 2 µM TDZ was higher than in other TDZ treatments (Table 1).

Amongst the three cytokinins used, TDZ induced a higher percentage of multiple shoot induction (59%) compared to that in BA (49.9%) and kinetin (44.2%) (Table 2). Of the five different concentrations used, 4 µM cytokinin induced the highest rate of shoot formation (64.4%) and the maximum number of shoots (6.6). The longest shoot length (3.4 cm) was attained on basal medium with 2 µM cytokinin (Table 2). These results suggest that increasing the cytokinin concentration beyond the optimum level decreases the rates of shoot initiation, multiplication, and elongation.

**Figure 1:**
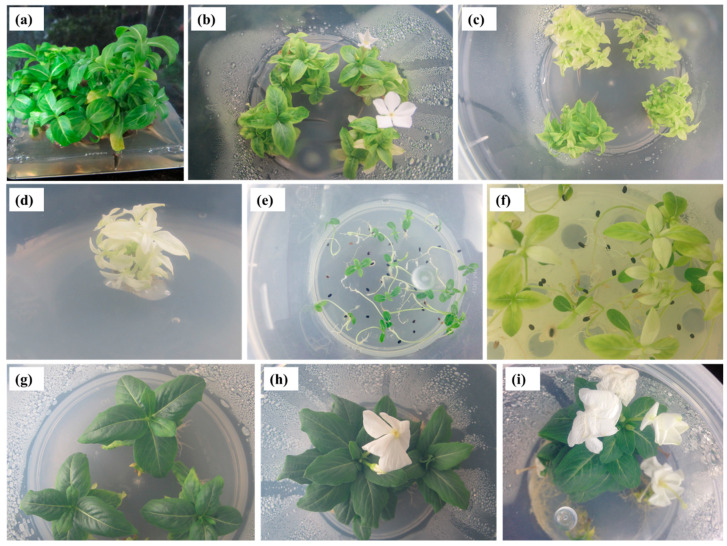
In vitro propagation of *Catharanthus roseus*. (**a**) Multiple shoots produced from nodal segments of *C. roseus* cultivated on MS medium with 2 µM TDZ and 1 µM NAA after 45 days; (**b**) in vitro flowers produced from the multiple shoots regenerated on MS medium with 2 µM TDZ, 1 µM NAA and 5% sucrose; (**c**) somaclonal variation in *C. roseus*; (**d**) multiple albino (variant) shoots produced from nodal segments isolated from the variant shoot cultivated on MS medium with 2 µM TDZ and 1 µM NAA; (**e**) seeds obtained from normal green plantlets were germinated on MS nutrient medium; (**f**) seeds obtained from somaclonal variant plantlets were germinated on MS nutrient medium; (**g**) root induction from a shoot cultivated on half-strength MS medium with 4 µM IBA; in vitro flowers produced from the rooted shoot cultivated on half-strength MS medium with 4 µM IBA plus (**h**) 3% sucrose and (**i**) 5% sucrose.

*C. roseus* nodal segments were inoculated on MS medium with 2, 4, or 8 µM TDZ and 0.5, 1, or 2 µM indole-3-acetic acid (IAA), indole-3-butyric acid (IBA), or NAA initiated shoot multiplication within a week of incubation. Both the TDZ and auxin levels were important for enhancing axillary shoot multiplication in *C. roseus*. The medium with 2 µM TDZ and 1 µM auxin (IAA, IBA, or NAA) had the best shoot induction percentages (Table 3). A higher shoot induction rate, higher number of shoots, and shoot growth were attained when the *C. roseus* nodal explants were cultivated on MS medium with 2 µM TDZ and 1 µM NAA (Figure 1a, Table 3). Lower shoot formation was observed on medium with higher TDZ levels with NAA, and callus induction was observed at the base of the *C. roseus* nodal explants. The highest rate of callus induction (100%) was obtained on medium with 8 µM TDZ and 2 µM NAA (data not shown).

Nodal explants developed shoots in the presence of sucrose (2–5%) and failed to produce shoots on the sucrose-free MS medium (Table 4). The highest shoot induction rate (95.8%) and highest number of shoots (23.6), with a mean length of 4.5 cm, were attained when the *C. roseus* nodal explants were cultivated on MS medium with 2 µM TDZ, 1 µM NAA, and 4% sucrose (Table 4). High sucrose concentrations (5%) inhibited the rate of shoot initiation and the number and length of induced axillary shoots. The shoots formed on MS medium containing 2 µM TDZ, 1 µM NAA, and sucrose (2–3%) failed to develop flowers after 45 days of cultivation. Higher sucrose concentrations (4 and 5%) promoted flowering within 30 days of incubation. The maximum rate of flowering (35.3%), with a mean of 2.9 flowers, was attained on MS medium containing 2 µM TDZ, 1 µM NAA, and 5% sucrose (Figure 1b, Table 4).

The number of subcultures had a significant effect on shoot multiplication and somaclonal variation in *C. roseus* (Table 5). The frequency of shoot induction increased with the number of subcultures, from zero to two, and then remained unchanged after six subcultures. The mean number of shoots increased up to three subcultures and significantly decreased thereafter (Table 3). The greatest number of shoots (39/nodal explant) was attained at the third subculture. Morphological changes in the shoots were observed after the third subculture; albino shoots were detected during the fourth subculture (Figure 1c), and the highest number of variant shoots (13.4) was attained at the sixth subculture (Table 5). The somaclonal variant shoots proliferated on MS medium with 2 µM TDZ and 1 µM NAA (Figure 1d) and were used for phytochemical analysis and biological assays. Seeds obtained from ex vitro acclimatized somaclonal variant and normal green plantlets were germinated on MS nutrient medium and displayed normal and variant shoots (Figure 1e,f).

The shoots developed roots after 14 days of culturing on half-strength MS medium containing 2–8 µM IBA (Table 6). The highest rooting response (90.9%) and highest number of roots (9.3), with a mean length of 6.2 cm, were attained on half-strength MS medium with 4 µM IBA after 35 days of culture (Figure 1g, Table 6). The lowest percentage of root induction (39.8%) was observed on half-strength MS medium with 4 µM IBA and no sucrose. Sucrose in the culture medium enhances the rooting response of shoots. However, the percentage of root induction and the number of roots varied with the concentration of sucrose (Table 7). The highest rate of root induction (96.7%) and number of roots (15.2), with a mean length of 8.3 cm, were observed on half-strength MS medium with 2% sucrose and 4 µM IBA. Higher sucrose concentrations (3–5%) reduced the percentage of root induction and the number of induced roots (Table 7). The in vitro-induced shoots (≥2 cm in length) developed on MS medium containing 2 µM TDZ and 1 µM NAA grew flowers within 20 days of cultivation on half-strength MS medium with 3–5% sucrose and 4 µM IBA (Figure 1h,i). The greatest rate of flowering (67.6%), with a mean number of 3.9 flowers, was obtained in a medium with 5% sucrose and 4 µM IBA after 35 days of cultivation (Table 7). The in vitro-developed *C. roseus* plantlets were acclimatized in a greenhouse with 98% survival; the acclimatized plants grew well without any morphological variations (data not shown).

### 2.2. Phytochemical Composition

The total content of phenolics and flavonoids in the extracts was measured using colorimetric methods (results shown in Table 8). The normal green shoots showed the highest level of phenolics (30.58 mg GAE/g), followed by the somaclonal variant shoots (26.45 mg GAE/g) and callus (14.66 mg GAE/g). The same order was observed for total flavonoids (normal green shoots (2.47 mg RE/g) > somaclonal variant shoots (1.21 mg RE/g) > callus (0.28 mg RE/g)).

Ultra-high-performance liquid chromatography–electrospray ionization–tandem mass spectrometry (UHPLC/ESI-MS/MS) was used for the rapid qualitative determination and identification of unknown compounds from different extracts and detailed results (retention time, protonated or deprotonated molecular ions, main fragment ions) are presented in Table 9, Table 10 and Table 11. MS/MS spectra contain rich structural information; however, because of the structural diversity of the molecules in the extracts, mass spectra were collected in positive and negative ionization modes separately. Some compounds were identified based on the retention times of the reference standards, protonated or deprotonated molecule ions, and characteristic fragment ions. In other cases, the unknown components were tentatively identified by their molecular ions and analyses of the UHPLC-MS/MS fragmentation data compared to published literature and/or our previous results (Figures S1–S9).

Fifty-five compounds were identified in the somaclonal variant shoot tissues, thirty-nine in the callus, and fifty-nine compounds in the normal green shoots. Similar components were found in the somaclonal variant and normal green shoot tissues.

Several groups of natural phenols, such as phenolic acids, *O*-caffeoylquinic acids, *O*-feruloylquinic acids, coumarin, quercetin, kaempferol, isorhamnetin derivatives, and other alkaloids, were identified in the samples. A wide range of low-molecular-weight polar compounds, e.g., methylcoumarin (MW: 178) and ajmalicine, a monomeric indole alkaloid (MW: 352), and higher molecular mass compounds, e.g., quercetin-O-dirhamnosylhexoside (MW: 756), were identified. Moreover, several known *Catharanthus* alkaloids, including vindolinine (Rt: 17.11 min), 19-*S*-vindolinine (Rt: 18.16 min), catharanthine (21.76 min), vindoline (Rt: 24.80 min), and vindolidine (Rt: 25.23 min), were chromatographically separated and characterized (Figures S10–S13).

### 2.3. Antioxidant Effects

The results are presented in Table 12. DPPH and ABTS were used to determine the scavenging ability of natural products or synthetics. As shown in Table 12, the normal green shoots exhibited better ability in both assays (IC50: 1.57 and 1.44 mg/mL for DPPH and ABTS, respectively). The weakest scavenging ability was observed in callus (IC50: >3 and 1.85 mg/mL for DPPH and ABTS, respectively). Similarly, the reducing power assays (CUPRAC and FRAP) indicated that the order of the samples was normal green shoots > somaclonal variant shoots > callus, reflecting the electron-donation abilities of the antioxidant compounds.

### 2.4. Enzyme Inhibitory Properties

We tested the enzyme inhibitory effects of *C. roseus* extracts against cholinesterases (AChE and BChE), tyrosinase, and amylase, and the results are reported in Table 13. The best AChE inhibitory effect was found in callus with an IC50 value of 0.65 mg/mL, followed by the normal green (IC50: 0.72 mg/mL) and somaclonal variant (IC50: 0.74 mg/mL) shoots. The samples had similar BChE inhibition values and the differences were non-significant. Similar results were also observed for amylase inhibition and the extracts exhibited close inhibition ability. As seen in Table 13, the best tyrosinase inhibitory effects were observed in the normal green (IC50: 0.83 mg/mL) and somaclonal variant shoots (IC50: 0.86 mg/mL).

## 3. Discussion

Multiple shoots initiated after nodal explants incubated on MS medium supplemented with cytokinins. The optimal BA concentration for axillary shoot multiplication from nodal explants of *C. roseus* was 4 µM (Table 1). The ability of BA to promote the formation of multiple *C. roseus* shoots was also observed in previous reports [19,25,26,28]. Pati et al. [19] reported that the nodal segments of *C. roseus* produced the maximum number of shoots (7.87) on MS liquid medium with 5 µM BA. In contrast, a growth medium containing 4.4 µM BA induced meager shoot formation (1.07) from nodal explants of *C. roseus* [25]. Amiri et al. [28] reported that the inclusion of 4.4 µM BA led to maximum shoot establishment (43%). However, 98% of the *C. roseus* nodal explants developed a mean of 7.12 shoots on MS medium with 4.4 µM BA [26]. These differences in shoot formation may be due to the different genotypes and explant sources. Kinetin also promotes multiple shoot production in *C. roseus* [19,24,26,27]. The percentage of shoot formation (59.4%), the number of shoots (5.7), and shoot elongation (3.6 cm) on MS medium with 4 µM kinetin were higher than in the other kinetin treatments and the control (Table 1). Similarly, Mehta et al. [26] reported that *C. roseus* nodal segments inoculated on medium with 4.4 µM kinetin developed 6.67 shoots, with a mean length of 2.7 cm. Pati et al. [19] reported that *C. roseus* single nodes inoculated in liquid medium with 5 µM kinetin formed 4.55 shoots, with an average length of 4.1 cm. Amongst the three cytokinins used, TDZ was the most effective in producing multiple shoots. TDZ, a plant growth regulator, has been shown to increase multiple shoot regeneration in a wide range of plants [29,30]. TDZ may enhance axillary shoot multiplication by varying the endogenous levels of growth regulators [30], and cytokinin concentration requirements differ for shoot induction and shoot elongation.

A combination of plant growth regulators (PGRs), such as cytokinin and auxin, was used to obtain a higher frequency of multiple shoot formation. Several studies have shown that media with cytokinin and auxin enhance shoot proliferation in *C. roseus* [9,16,25,26,28]. Satdive et al. [16] reported that the morphogenetic response (73.33%) and the number of shoots (9–13 per cotyledonary leaf) were highest in medium with 11.4 µM kinetin and 0.27 µM NAA. Kumar et al. [25] reported that the multiplication rate (4.01 shoots/nodal segment) and shoot length (2.07 cm) were highest in medium with 4.4 µM BA and 1.08 µM NAA, and Mehta et al. [26] reported that the shooting response (99%), number of shoots (7.3/node), and shoot length (5.97 cm) were highest with 2.2 µM BA and 10.8 µM NAA. Amiri et al. [28] reported that the multiplication rate (5.2 shoots/nodal segment) and shoot length (6.3 cm) were highest with 6.6 µM BA and 2.5 µM IBA. Although TDZ has both auxin and cytokinin activities, the addition of TDZ medium with auxin often improves the in vitro shoot production of a wide range of plants [29,30,46]. In this study, a higher shoot induction rate (91.1%) and higher number of shoots (19.2), with a mean length of 4.9 cm, were attained when the *C. roseus* nodal explants were cultivated on MS medium with 2 µM TDZ and 1 µM NAA (Table 3).

The impact of sucrose on multiple shoot production in *C. roseus* is unreported. In this study, sucrose had a significant effect on multiple shoot formation. The supplementation of sucrose or sugar is essential to stimulate axillary bud growth in vitro [47]. Sucrose in the cultivation media may increase the endogenous levels of carbohydrates, such as sucrose, glucose, fructose, and starch [48,49], and plant hormones, such as IAA, isopentenyl adenine riboside 5′-monophosphate, isopentenyl adenine riboside, isopentenyl adenine, zeatin riboside 5′-monophosphate, and zeatin riboside [50], that are important for various phases of plant growth. Starch accumulation is a prerequisite for shoot initiation in numerous plants [51]. Endogenous glucose levels improve the PGR-induced growth response. Glucose may affect the auxin biosynthetic YUCCA gene family members, auxin transporter PIN proteins, receptor TIR1, and the members of several gene families, including AUX/IAA, GH3, and SAUR, that are involved in auxin signaling [52]. Genes involved in cytokinin biosynthesis, such as AHK2, AtCKX4, AtCKX5, AtHXK4, ARR10, ARR1, ARR2, ARR6, ARR8, ARR11, CRF1, CRF2, CRF3, and IP3, are also regulated by glucose [53]. The highest multiple shoot production was attained when the *C. roseus* nodal explants were cultivated on MS medium with 2 µM TDZ, 1 µM NAA, and 4% sucrose (Table 4). However, the presence of 5% sucrose inhibited the rate of shoot initiation (67.8%). Sucrose, either alone or via interaction with other plant hormones, can induce or suppress many of the growth-related genes [50,54], which subsequently enhances or reduces the shooting response.

Flowering is regulated by internal plant factors and environmental signals [55]. The in vitro flower induction depends on culture environment, PGRs, media composition, and sucrose level [56]. The in vitro flowering of *C. roseus* is also affected by sucrose (Table 4), which promotes in vitro flowering in many plants [40,57,58]. Recently, *C. roseus* in vitro flowering has been achieved by using silver nitrate [27]. However, to our knowledge, the influence of sucrose on the in vitro flowering of *C. roseus* is unreported. In this study, including 4% and 5% sucrose in the MS nutrient medium promoted flowering in *C. roseus* (Table 4). Similar results have been reported for *Ceropegia rollae* [58], *Scrophularia takesimensis* [40], and *Withania somnifera* [57]. The in vitro flowering procedure established in this study can be utilized in bioactive compounds, mainly alkaloid [27] production, and in vitro breeding of *C. roseus*.

The continuous exposure of explants to shoot induction medium during several subcultures decreased the morphogenetic potential (Table 5). Thus, a secondary medium (TDZ-free MS) was required to maintain the morphogenetic potential of the nodal explants, where multiple shoots induced after the third subculture were elongated (Table 5). Several studies have shown that TDZ is slowly metabolized by plants and affects shoot formation [30,32]. The adverse effects of continued TDZ presence on shoot multiplication have also been reported in several plants [29,30]. In this study, somaclonal variants (albino shoots) were detected during the fourth subculture. Continuous exposure to TDZ also resulted in leaf chlorosis in *Astragalus schizopterus* [59], *Philodendron cannifolium* [60], and *Sphagneticola trilobata* [61]. Dewir et al. [32] reported that the TDZ-induced SV may be a valuable source of new genetic material. In this study, seeds obtained from the somaclonal plantlets were successfully germinated on MS nutrient medium and several seedlings exhibited a similar morphology. The somaclonal variants obtained in this study will be useful for new cultivar development.

There was no root formation in the absence of IBA; similar results have been reported in *C. roseus* [19,28]. Rooting of the in vitro-developed shoots of *C. roseus* was observed on auxin-free medium [25,26,36]. Differences in the rooting ability of micro shoots may be due to the endogenous levels of PGRs. When cytokinins were applied to induce shoot multiplication, they often inhibited the subsequent rooting of in vitro-regenerated shoots [19,28]. The rooting ability of micro shoots also depends on the type and concentration of cytokinins used in the shoot induction medium. TDZ has high cytokinin activity and strongly inhibits the activity of cytokinin oxidase, which increases the endogenous levels of natural cytokinins [29]. Thus, TDZ inhibits adventitious root formation. IBA has also been used for in vitro rooting in *C. roseus* [19,25,26]. In this study, higher IBA concentrations (12 µM) significantly diminished the rate of rooting (26.7%), number of roots (2.9), and elongation of the roots (2.1 cm) (Table 6). This is consistent with an earlier study of *C. roseus* [25]. In contrast, the highest rooting response (80%) and number of roots (7.0), with a mean length of 1.66 cm, were achieved in MS liquid medium containing 10 µM IBA [19]. The highest rate of root induction (90%) and number of roots (3.6), with a mean length of 1.68 cm, were achieved in quarter-strength MS medium with 24.6 µM IBA [26]. Root formation is an energy-consuming process that requires a source of carbon [62]. Sucrose is an important sugar that is frequently used in plant tissue culture medium as a source of energy and osmoticum. A culture medium with low osmotic potential is often preferred for the induction of roots and the osmotic potential is mostly maintained by sucrose. Low sucrose concentrations (2%) in the medium may decrease the osmotic potential and improve the rooting response of *C. roseus* (Table 7).

Different results have been reported in previous studies evaluating the total bioactive compounds of *C. roseus*. These differences may be explained based on differences in culture conditions, harvest times, or mineral intake [8,63]. Nonetheless, spectrophotometric methods have some drawbacks. For example, phenolics and other compounds (e.g., proteins) could interact with the Folin–Ciocalteu reagent and interfere with the results [64]. Moreover, some phytochemicals may form a complex with AlCl3 [65]. Thus, the identification, qualification, and quantification of phytochemicals should be confirmed using chromatographic methods, such as high-performance liquid chromatography (HPLC), nuclear magnetic resonance (NMR), and gas chromatography (GC), for more accurate results. In this study, the phytochemicals in *C. roseus* extracts were qualified using UHPLC-MS/MS.

The term “antioxidant” has gained interest because it may play a role in preventing chronic and degenerative diseases. Several investigations have suggested that an imbalance between oxidants and antioxidants is the main reason for disease progression. Thus, many attempts have been made to find novel and safe antioxidants, and most have involved plants or plant products [66]. In light of the facts mentioned above, the antioxidant properties of the *C. roseus* samples were tested via different chemical methods, including free radical scavenging, reducing power, metal chelating, and phosphomolybdenum. In this study, the best antioxidant properties were obtained in the normal green shoot, followed by somaclonal variant shoot and callus extracts. These results can be attributed to the levels of phenolics in the extracts, as suggested by several researchers [67,68], who reported a positive correlation between the concentration of phenolics and their antioxidant properties. The metal chelating ability was ranked as somaclonal variant shoots > normal green shoots > callus. These contradictory results may be due to the non-phenolic chelators in the somaclonal variant shoots [5]. Some authors have also suggested that metal chelation plays a minor role in the antioxidant abilities of phenolic compounds. Studies on the antioxidant properties of *C. roseus* have yielded variable results. For example, Moon et al. [63] reported that the reducing power activity (FRAP and CUPRAC assays) of *C. roseus* samples was affected by culture conditions. Pham et al. [8] investigated the bioactivity and observed activity of *C. roseus* stem extracts and found that they were dependent on the solvents used. Finally, Pereira et al. [69] grew *C. roseus* roots in a 25 °C growth chamber for 16 h, and the root extracts exhibited significant free radical scavenging abilities in the in vitro assays. Taken together, these results suggest that *C. roseus* may be a natural raw material for novel antioxidants in the pharmaceutical and nutraceutical industries.

In the 21st century, some diseases are considered epidemiological pandemics and have created global crises. Alzheimer’s disease, diabetes mellitus, and obesity are such diseases [70,71], which require effective therapeutic strategies. One of the approaches to tackling this issue is the inhibition of enzymes that play roles in disease progression. Keeping this in mind, several key enzymes have been targeted. Carbohydrate-hydrolyzing enzymes (amylase and glucosidase) are the main targets for managing and preventing diabetes mellitus; their inhibition could retard the increase in blood glucose levels after a carbohydrate-rich diet [72]. Cholinesterases (especially acetylcholinesterase) are important factors in neurotransmission across synaptic gaps, and the inhibition of these may enhance the cognitive functioning in patients with Alzheimer’s disease [73]. Based on these facts, some compounds are produced as effective inhibitors in the pharmaceutical industry. However, most of these compounds have undesirable side effects [72,74]. Thus, novel and safe inhibitors from natural sources are needed to ameliorate the above-mentioned diseases. In the present study, the enzyme inhibitory effects of *C. roseus* extracts were investigated using different enzymes. We observed different results for each enzyme inhibition ability. To date, there have been few reports on the enzyme inhibitory effects of *C. roseus*. Pereira et al. [75] reported significant inhibitory effects of *C. roseus* root alkaloids against acetylcholinesterase, and vindoline and serpentine exhibited good anti-cholinesterase inhibition effects. These alkaloids were also found in our study and the combined results suggested that the cholinesterase inhibitory effects may be due to the presence of these alkaloids. Several other researchers have also reported alkaloids as effective inhibitors of cholinesterases. Moreover, some of the alkaloids from *C. roseus* exhibit significant antidiabetic effects in vivo. Tyrosinase is the main enzyme of melanin synthesis and is important for controlling hyperpigmentation problems [76]. In this study, the best tyrosinase inhibitory effect was detected in the normal green shoot extracts of *C. roseus*. From a pharmacological perspective, *C. roseus* may be an effective weapon against global health problems.

## 4. Materials and Methods

### 4.1. In Vitro Micropropagation

#### 4.1.1. Plant Materials and Surface Decontamination

Actively growing shoots were collected from 6-month-old *C. roseus* plants cultivated in a greenhouse. The shoots were thoroughly rinsed under running tap water for 20 min, soaked in Tween 20 (0.1%, *v*/*v*) for 12 min, and then rinsed with distilled water. The shoots were surface decontaminated in 70% (*v*/*v*) ethanol (Daejung, Siheung-si, Gyeonggi-do, Korea) for 30 s, 5% (*v*/*v*) sodium hypochlorite (Daejung, Siheung-si, Gyeonggi-do, Korea) solution containing 3–6 drops of Tween 20 for 15 min, and 70% ethanol for 60 s. Each treatment was followed by 3–5 rinses using sterilized distilled water containing 0.1% (*w*/*v*) polyvinylpyrrolidone (Duchefa, Haarlem, The Netherlands).

#### 4.1.2. Axillary Shoot Multiplication

The decontaminated shoots were cut into single nodal segments (0.6–1 cm) cultured in MS [77] medium fortified with 0, 1, 2, 4, 8, or 16 µM BA, kinetin, or TDZ and 2, 4, or 8 µM TDZ plus 0.5, 1, or 2 µM 2-(1-naphthyl) acetic acid (NAA), indole-3-butyric acid (IBA), or indole-3-acetic acid (IAA) for axillary shoot multiplication. To study the effects of sucrose on multiple shoot induction and flowering, nodal explants were inoculated on MS medium with optimal plant growth regulators (2 µM TDZ and 1 µM NAA) plus 0, 2, 3, 4, or 5% (*w*/*v*) sucrose. To study the effects of subculturing on shoot multiplication and SV, nodal explants derived from the in vitro multiple shoots (each subculture) were inoculated on MS medium with the optimal plant growth regulators and 4% sucrose. The shoot induction medium consisted of MS basal nutrients and vitamins with 3% sucrose (unless otherwise specified) and solidified with 0.8% (*w*/*v*) plant agar. The pH of the cultivation medium was adjusted to 5.6–5.8 before autoclaving at 121 °C for 20 min. The cultures were kept for 45 days at 23 ± 1 °C in a 16/8 light/dark photoperiod (50 µmol m^−2^ s^−1^), provided by cool white fluorescent tubes. The experiments were conducted as a completely randomized design; ten explants were used in each treatment, with three replications, and all experiments were performed twice. The shoot induction rate, total number of shoots, shoot length, percentage of flowering, total number of flowers, and total number of variant shoots were assessed after 45 days.

#### 4.1.3. Rooting and Acclimatization

For root induction, in vitro-induced shoots (≥2 cm in length) were separated from the shoot clusters and inoculated on 1/2 MS medium with 0, 1, 2, or 4 µM IBA. To study the effects of sucrose on root induction and flowering, shoots were cultured on 1/2 MS medium fortified with 0, 2, 3, 4, or 5% sucrose and 4 µM IBA. For acclimatization, the rooted shoots were removed from the 1/2 MS medium, rinsed in tap water, and transplanted into plastic cups (200 mL) containing autoclaved peat moss, perlite, and vermiculite (1:1:1, *v*/*v*/*v*). The shoots were irrigated at four-day intervals with a 1/4 MS basal nutrient solution. The experiments were conducted as a completely randomized design; ten explants were used in each treatment, with three replications, and all experiments were performed twice. The rate of root induction, total number of roots, root length, percentage of flowering, total number of flowers, and plantlet survival were recorded after 35 days. The data were subjected to analysis of variance tests (ANOVA) in SAS (Release 9.1, SAS Institute, NC, USA).

### 4.2. Phytochemical Analysis

#### 4.2.1. Extract Preparation

Callus (obtained from MS with 8 µM TDZ and 2 µM NAA), somaclonal variant, and normal green shoots (collected from MS with 2 µM TDZ and 1 µM NAA) were obtained from 45-day-old in vitro cultures, cut into small pieces, stored at −70 °C for 16 h, and then lyophilized. The freeze-dried samples (0.5 g) were extracted with methanol (80%) using an ultraturrax at 6000× *g* for 20 min. The extracts were filtered, and the solvents were removed using a rotary evaporator. All extracts were stored at 4 °C until further analysis.

#### 4.2.2. Identification and Quantification of the Phytochemicals

Gradient reversed-phase ultra-high-performance liquid chromatography (UHPLC) separations with electrospray tandem mass spectrometry (MS/MS) detection (both positive and negative ion modes) were used for the structural characterization of the compounds in the extracts. The UHPLC system consisted of a Dionex Ultimate 3000RS UHPLC instrument coupled to a Thermo Q Exactive Orbitrap mass spectrometer. Chromatographic separation was achieved on a reversed-phase column Thermo Accucore C18 (100 mm × 2.1 mm i.d., 2.6 µm) [78]. Analytical details are presented in the Supplementary Materials.

#### 4.2.3. Determination of Total Phenolics and Flavonoids

The total phenolic content was determined via the Folin–Ciocalteu method, as described by Slinkard and Singleton [79], and calculated as the gallic acid equivalent (GAE). The total flavonoid content was determined using the aluminum chloride (AlCl3) method, according to Zengin et al. [80], and was expressed as the rutin equivalent (RE).

### 4.3. Biological Activities

#### 4.3.1. Antioxidant Activity

The antioxidant potential of the extracts was measured using several assay models, as previously described by Uysal et al. [81]. These include the radical scavenging assays for ABTS (2,2′-azino-bis(3-ethylbenzothiazoline-6-sulphonic acid) and DPPH (2,2-diphenyl-1-picrylhydrazyl) radicals, the redox assays for FRAP (ferric reducing antioxidant power) and CUPRAC (cupric reducing antioxidant capacity), and phosphomolybdenum total antioxidant capacity (TAC). Metals may catalyze the oxidation reactions; therefore, a metal chelating assay was also performed. Trolox and EDTA (for the chelating assay) were used as reference antioxidant compounds.

#### 4.3.2. Enzyme Inhibition Assay

The extracts were tested for possible enzyme inhibition activity against several drug targets of different human diseases. Their activity was expressed in comparison to known drug inhibitors; acarbose for amylase, galantamine for acetylcholinesterase (AChE) and butylcholinestrase (BChE), and kojic acid for tyrosinase. All assay procedures were conducted according to methods described by Uysal et al. [81].

## 5. Conclusions

A competent in vitro propagation system through axillary shoot multiplication was established for C. roseus. This study showed that the PGRs and sucrose are significant factors affecting shoot bud initiation and multiplication from nodal segments. High levels of sucrose in the shoot induction or rooting medium have positive effects on in vitro flowering. SV was observed after the third subculture. In vitro flowering and SV may be exploited for *C. roseus* improvement. Phytochemical analysis indicated the presence of several phenolics and alkaloids in the callus, normal green, and somaclonal variant shoot extracts of *C. roseus*. Additionally, the extracts possessed potent antioxidant and enzyme inhibitory activities. These findings suggest that in vitro-derived callus, somaclonal variant, and normal green shoots may serve as alternative sources of bioactive metabolites with antioxidant and enzyme inhibitory activities. However, further experimental studies, such as in vivo animal models and toxicological assays, are recommended.

## Figures and Tables

**Table 1:** Effect of cytokinins on multiple shoot regeneration from nodal explants of *Catharanthus roseus.*

Cytokinin	Conc. (µM)	Shoot Induction (%)	Shoot Number	Shoot Length (cm)
Control (MS)	0	18.3 ± 2.7 j	1.3 ± 0.3 i	1.0 ± 0.5 h,i
BA	1	23.1 ± 4.8 i	2.6 ± 0.7 h	2.5 ± 0.4 d-f
	2	55.8 ± 3.9 d	4.0 ± 1.0 e,f	3.1 ± 0.4 d
	4	66.4 ± 3.7 b	6.3 ± 1.3 c	2.9 ± 0.6 d,e
	8	60.3 ± 2.9 c	3.7 ± 1.0 e-g	2.4 ± 0.4 e-g
	16	44.2 ± 4.0 f	2.7 ± 1.2 g,h	1.5 ± 0.3 h
Kinetin	1	25.7 ± 2.8 i	2.3 ± 0.9 h	1.8 ± 0.5 g,h
	2	32.7 ± 2.8 h	3.1 ± 0.8 f-h	2.1 ± 0.4 f,g
	4	59.5 ± 2.4 c	5.7 ± 1.0 c	3.6 ± 0.8 c
	8	54.2 ± 3.0 d	4.4 ± 1.1 d,e	2.2 ± 0.4 f,g
	16	48.7 ± 3.3 e	2.9 ± 1.1 g,h	1.5 ± 0.3 h
TDZ	1	37.8 ± 2.8 g	4.3 ± 0.9 d,e	4.4 ± 1.1 b
	2	75.2 ± 3.7 a	10.1 ± 1.5 a	5.0 ± 0.7 a
	4	67.1 ± 3.4 b	7.7 ± 1.2 b	3.0 ± 0.4 d
	8	61.7 ± 5.5 c	5.3 ± 0.7 c,d	1.4 ± 0.3 h
	16	53.3 ± 3.1 d	3.7 ± 1.0 e-g	0.8 ± 0.2 i
		*f*-value		
F-test	Cytokinin	196.6	83.0	18.7
	Conc.	393.4	60.5	73.8
	Cytokinin * Conc.	50.8	15.8	30.8
		*p*-value		
	Cytokinin	<0.001	<0.001	<0.001
	Conc.	<0.001	<0.001	<0.001
	Cytokinin * Conc.	<0.001	<0.001	<0.001

Mean ± S.D., followed by the same letters within a column, were not significantly different *p* < 0.05. BA, N^6^-benzyladenine; TDZ, thidiazuron; Conc., concentration.

**Table 2:** Effect of cytokinins and their concentration on multiple shoot regeneration from nodal explants of *Catharanthus roseus*.

Factors	Shoot Induction (%)	Shoot Number	Shoot Length (cm)
BA	49.9 ± 17.1 b	3.8 ± 1.5 b	2.5 ± 0.6 b
Kinetin	44.2 ± 14.4 c	3.7 ± 1.4 b	2.2 ± 0.8 b
TDZ	59.0 ± 14.3 a	6.2 ± 2.7 a	2.9 ± 1.8 a
1 µM	28.9 ± 7.8 e	3.1 ± 1.1 d	2.9 ± 1.3 b
2 µM	54.6 ± 21.3 c	5.7 ± 3.8 b	3.4 ± 1.5 a
4 µM	64.4 ± 4.2 a	6.6 ± 1.0 a	3.1 ± 0.4 ab
8 µM	58.7 ± 4.0 b	4.5 ± 0.8 c	1.9 ± 0.5 c
16 µM	48.7 ± 4.6 d	3.1 ± 0.5 d	1.3 ± 0.4 d

Mean ± S.D., followed by the same letters within a column, were not significantly different *p* < 0.05. The means in Table 2 refer to all concentrations and effects observed in Table 1. BA, N^6^-benzyladenine; TDZ, thidiazuron.

**Table 3:** Effect of TDZ plus auxins on multiple shoot induction from nodal explants of *Catharanthus roseus*.

Concentration (µM)				Shoot Induction (%)	Shoot Number	Shoot Length (cm)
TDZ	IAA	IBA	NAA			
0	0	0	0	18.3 ± 2.7 r	1.3 ± 0.3 r	1.0 ± 0.5 k
2	0.5	0	0	79.1 ± 2.4 c,d	11.0 ± 1.2 e,f	3.8 ± 0.5 c,d
4	0.5	0	0	69.2 ± 3.4 h-k	8.0 ± 1.7 j-l	3.3 ± 0.6 e,f
8	0.5	0	0	66.3 ± 3.3 k,l	5.2 ± 1.3 n-p	1.7 ± 0.5 j
2	1	0	0	82.3 ± 2.7 b,c	12.9 ± 1.5 c,d	4.1 ± 0.6 b,c
4	1	0	0	80.4 ± 4.7 c	10.0 ± 1.7 f,g	2.8 ± 0.4 g
8	1	0	0	70.1 ± 3.5 g-j	7.3 ± 1.2 k-m	1.9 ± 0.4 h-j
2	2	0	0	63.6 ± 3.2 l,m	8.9 ± 1.6 g-j	3.0 ± 0.2 f,g
4	2	0	0	43.7 ± 3.2 p	6.3 ± 0.9 m,n	2.8 ± 0.3 g
8	2	0	0	38.2 ± 3.5 q	4.1 ± 1.2 p,q	1.1 ± 0.3 k
2	0	0.5	0	80.8 ± 4.1 c	13.3 ± 1.7 c,d	4.5 ± 0.4 b
4	0	0.5	0	70.9 ± 2.9 g-i	9.4 ± 1.3 f-j	3.6 ± 0.3 d,e
8	0	0.5	0	67.3 ± 4.4 j,k	7.1 ± 1.5 k-m	1.8 ± 0.2 i,j
2	0	1	0	84.1 ± 2.8 b	14.9 ± 1.8 b	4.1 ± 0.3 b,c
4	0	1	0	76.4 ± 3.4 d,e	10.4 ± 1.2 f,g	4.4 ± 0.5 b
8	0	1	0	73.1 ± 3.6 f,g	8.1 ± 1.1 i-l	2.3 ± 0.3 h
2	0	2	0	68.2 ± 3.5 i-k	6.8 ± 1.2 l,m	3.3 ± 0.4 e,f
4	0	2	0	60.4 ± 2.2 m,n	5.9 ± 0.8 n-o	2.2 ± 0.4 h,i
8	0	2	0	45.8 ± 3.8 p	4.7 ± 1.2 o-q	1.0 ± 0.3 k
2	0	0	0.5	79.0 ± 3.3 c,d	14.3 ± 1.7 b,c	5.1 ± 0.3 a
4	0	0	0.5	75.2 ± 3.7 e,f	10.2 ± 1.6 f,g	4.4 ± 0.5 b
8	0	0	0.5	67.1 ± 4.0 j,k	8.4 ± 1.7 h-k	2.8 ± 0.4 g
2	0	0	1	91.1 ± 2.7 a	19.2 ± 2.0 a	4.9 ± 0.4 a
4	0	0	1	79.3 ± 2.9 c,d	12.3 ± 2.1 d,e	3.5 ± 0.5 d,e
8	0	0	1	69.9 ± 3.6 g-j	9.7 ± 1.9 f-i	2.7 ± 0.3 g
2	0	0	2	72.6 ± 2.6 f-h	9.6 ± 1.9 f-j	3.3 ± 0.4 e,f
4	0	0	2	60.1 ± 3.3 g-j	9.5 ± 1.8 f-j	2.1 ± 0.2 h,i
8	0	0	2	52.7 ± 2.7 o	3.2 ± 1.6 q	1.1 ± 0.3 k

Mean ± S.D., followed by the same letters within a column, were not significantly different *p* < 0.05. TDZ, thidiazuron; IAA, indole-3-acetic acid; IBA, indole-3-butyric acid; NAA, 2-(1-Naphthyl)acetic acid.

**Table 4:** Effect of sucrose on in vitro multiple shoot induction and flowering of Catharanthus roseus.

Sucrose (%)	Shoot Induction (%)	Shoot Number	Shoot Length (cm)	Flowering (%)	Flower Number
0	0.0 ± 0.0 e	0.0 ± 0.0 e	0.0 ± 0.0 d	0.0 ± 0.0 c	0.0 ± 0.0 c
2	85.6 ± 2.9 c	13.7 ± 1.3 d	3.6 ± 0.5 c	0.0 ± 0.0 c	0.0 ± 0.0 c
3	91.1 ± 2.7 b	19.2 ± 2.0 b	4.9 ± 0.4 a	0.0 ± 0.0 c	0.0 ± 0.0 c
4	95.8 ± 2.0 a	23.6 ± 2.7 a	4.5 ± 0.4 b	23.8 ± 3.7 b	2.0 ± 0.7 b
5	67.8 ± 3.4 d	15.8 ± 2.3 c	3.9 ± 0.7 c	35.3 ± 4.0 a	2.9 ± 1.2 a

Mean ± S.D., followed by the same letters within a column, were not significantly different *p* < 0.05. Medium: Murashige and Skoog with 2 µM thidiazuron and 1 µM 2-(1-Naphthyl)acetic acid.

**Table 5:** Effect of subculture on shoot multiplication and somaclonal variation in *Catharanthus roseus*.

No. of Subculture	Shoot Induction (%)	Normal Shoot Number	Variant Shoot Number
0	95.8 ± 2.0 c	23.6 ± 2.7 d	0.0 ± 0.0 d
1	98.3 ± 1.5 b	28.3 ± 2.7 c	0.0 ± 0.0 d
2	100 ± 0.0 a	31.7 ± 3.3 b	0.0 ± 0.0 d
3	100 ± 0.0 a	39.0 ± 2.9 a	0.0 ± 0.0 d
4	100 ± 0.0 a	20.4 ± 2.6 e	7.4 ± 1.7 c
5	100 ± 0.0 a	14.8 ± 1.9 f	11.8 ± 1.6 b
6	100 ± 0.0 a	7.9 ± 1.1 g	13.4 ± 2.1 a

Mean ± S.D., followed by the same letters within a column, were not significantly different *p* < 0.05. Medium: Murashige and Skoog with 2 µM thidiazuron and 1 µM 2-(1-Naphthyl)acetic acid and 4% sucrose.

**Table 6:** Effect of IBA on in vitro rooting of *Catharanthus roseus*.

IBA (µM)	Rooted Shoot (%)	Number of Roots	Root Length (cm)
0	0.0 ± 0.0 e	0.0 ± 0.0 e	0.0 ± 0.0 d
2	57.7 ± 6.3 c	5.1 ± 1.4 c	3.5 ± 0.8 b
4	90.9 ± 5.2 a	9.3 ± 1.3 a	6.2 ± 1.8 a
8	78.4 ± 6.2 b	6.4 ± 1.4 b	5.2 ± 1.1 a
12	26.7 ± 4.9 d	2.9 ± 1.1 d	2.1 ± 0.8 c

Mean ± S.D., followed by the same letters within a column, were not significantly different *p* < 0.05. Medium: Half-strength Murashige and Skoog with 3% sucrose. IBA, indole-3-butyric acid.

**Table 7:** Effect of sucrose on in vitro rooting and flowering of *Catharanthus roseus*.

Sucrose (%)	Rooted Shoot (%)	Number of Roots	Root Length (cm)	Flowering (%)	Flower Number
0	39.8 ± 7.2 e	2.7 ± 1.2 e	2.9 ± 0.9 d	0.0 ± 0.0 d	0.0 ± 0.0 d
2	96.7 ± 3.8 a	15.2 ± 2.5 a	8.3 ± 1.4 a	0.0 ± 0.0 d	0.0 ± 0.0 d
3	90.9 ± 5.2 b	9.3 ± 1.3 b	6.2 ± 1.9 b	32.6 ± 9.6 c	1.3 ± 0.5 c
4	79.6 ± 5.7 c	6.1 ± 1.9 c	4.9 ± 1.5 bc	56.4 ± 7.9 b	2.3 ± 0.7 b
5	67.3 ± 6.2 d	4.4 ± 1.3 d	4.4 ± 1.7 c	67.6 ± 6.2 a	3.9 ± 1.2 a

Mean ± S.D., followed by the same letters within a column, were not significantly different *p* < 0.05. Medium: Half-strength Murashige and Skoog with 4 µM indole-3-butyric acid.

**Table 8:** Total phenolic and flavonoid content in the extracts.

	Total Phenolic Content (mg GAE/g)	Total Flavonoid Content (mg RE/g)
Somaclonal variant shoot	26.45 ± 0.17	1.21 ± 0.07
Callus	14.66 ± 0.06	0.28 ± 0.09
Normal green shoot	30.58 ± 0.66	2.47 ± 0.07

Values are expressed as mean ± S.D. of three parallel measurements. GAE, gallic acid equivalent; RE, rutin equivalent.

**Table 9:** Chemical composition of somaclonal variant shoot tissues of *Catharanthus roseus*.

No.	Name	Formula	Rt	[M + H]^+^	[M – H]^−^	Fragment 1	Fragment 2	Fragment 3	Fragment 4	Fragment 5	References
1	Neochlorogenic acid (5-*O*-Caffeoylquinic acid)	C_16_H_18_O_9_	10.12	355.10291		163.0387	145.0283	135.0440	117.0337	89.0389	
2 ^1^	Chlorogenic acid (3-*O*-Caffeoylquinic acid)	C_16_H_18_O_9_	14.85	355.10291		163.0388	145.0283	135.0440	117.0335	89.0389	
3	3-*O*-Feruloylquinic acid cis isomer	C_17_H_20_O_9_	14.87		367.10291	193.0498	191.0550	173.0443	134.0362		
4	3-*O*-Feruloylquinic acid	C_17_H_20_O_9_	15.11		367.10291	193.0498	191.0556	173.0443	134.0360	93.0330	
5	Methylcoumarin isomer 1	C_10_H_8_O_2_	15.71	161.06026		133.0647	105.0701	103.0545	91.0545	79.0547	
6	Loganic acid	C_16_H_24_O_10_	15.72		375.12913	213.0761	169.0858	151.0753	113.0229	69.0329	
7	Chryptochlorogenic acid (4-*O*-Caffeoylquinic acid)	C_16_H_18_O_9_	16.09	355.10291		163.0387	145.0283	135.0440	117.0336	89.0388	
8	Vindolinine	C_21_H_24_N_2_O_2_	17.11	337.19161		320.1641	276.1383	177.0909	144.0807	117.0700	[42,43]
9	Secologanoside	C_16_H_22_O_11_	17.27		389.10839	345.1190	209.0448	165.0545	121.0644	69.0329	
10	Unidentified alkaloid	C_20_H_24_N_2_O_2_	18.08	325.19161		307.1801	277.1320	186.0914	174.0912	138.0913	
11	19-*S*-Vindolinine	C_21_H_24_N_2_O_2_	18.16	337.19161		320.1640	276.1380	177.0908	144.0807	117.0700	[42,43]
12	Unidentified alkaloid	C_20_H_22_N_2_O_2_	18.17	323.7596		248.1437	219.1039	173.1072	144.0807	79.0548	
13	Dihydrositsirikine	C_21_H_28_N_2_O_3_	18.37	357.21782		339.2061	311.1382	251.1178	234.0910	136.1120	[44]
14	5-*O*-Feruloylquinic acid	C_17_H_20_O_9_	18.47		367.10291	193.0499	191.0552	173.0442	134.0360	93.0329	
15	Unidentified alkaloid	C_21_H_28_N_2_O_3_	18.94	357.21782		253.1694	226.1434	214.1435	144.0807	110.0966	
16	4-*O*-Feruloylquinic acid	C_17_H_20_O_9_	18.99		367.10291	193.0498	191.0551	173.0443	134.0360	93.0329	
17	Loganin	C_17_H_26_O_10_	19.05	391.16043		229.1067	197.0820	179.0703	151.0752	109.0649	[45]
18	Methylcoumarin isomer 2	C_10_H_8_O_2_	19.06	161.06026		133.0648	105.0702	103.0545	91.0547	79.0546	
19	Unidentified alkaloid	C_20_H_22_N_2_O_2_	19.67	323.17596		216.1017	184.0758	156.0807	129.0699		
20	Antirhine isomer	C_19_H_24_N_2_O	19.83	297.19669		280.1698	236.1428	166.1221	154.1225	144.0807	
21	11-Hydroxycyclolochnerine or Lochneridine	C_20_H_24_N_2_O_3_	19.91	341.18652		323.1751	281.1640	264.1386	218.0808	200.0703	[44]
22	Vinervine	C_20_H_22_N_2_O_3_	20.07	339.17087		307.1435	279.1484	250.1215	185.0704		
23	Panarine	C_20_H_22_N_2_O_2_	20.32	323.17596		305.1643	166.0860	156.0804	148.1119	144.0806	
24	Secologanol	C_17_H_26_O_10_	20.36	391.16043		229.1068	211.0963	193.0859	179.0701	167.0702	
25	5-*O*-Feruloylquinic acid cis isomer	C_17_H_20_O_9_	20.51		367.10291	193.0490	191.0552	173.0445	134.0360	93.0330	
26	Ammocalline	C_19_H_22_N_2_	20.64	279.18612		248.1431	219.1039	149.0232	144.0807	107.0858	[44]
27	Antirhine	C_19_H_24_N_2_O	20.82	297.19669		280.1689	196.1122	166.1224	154.1225	144.0807	[44]
28	Unidentified alkaloid	C_21_H_24_N_2_O_2_	20.95	337.19161		305.1639	222.1276	180.1018	156.0806	144.0807	
29	Quercetin-*O*-dirhamnosylhexoside	C_33_H_40_O_20_	21.05		755.20347	301.0354	300.0275	299.0198	271.0247	255.0296	
30	Cathenamine or Vallesiachotamine	C_21_H_22_N_2_O_3_	21.26	351.17087		321.1592	289.1330	247.1226	233.1069	182.0836	[44]
31	11-Hydroxycyclolochnerine or Lochneridine	C_20_H_24_N_2_O_3_	21.48	341.18652		323.1749	279.1491	264.1381	198.0913	138.1277	[44]
32	Cathenamine or Vallesiachotamine	C_21_H_22_N_2_O_3_	21.59	351.17087		321.1590	289.1333	247.1225	196.0752	168.0805	[44]
33	Akuammicine	C_20_H_22_N_2_O_2_	21.60	323.17596		294.1484	291.1487	280.1330	263.1538	234.1279	[44]
34	Catharanthine	C_21_H_24_N_2_O_2_	21.76	337.19161		173.1071	165.0907	144.0806	133.0648	93.0702	[43,45]
35	Ajmalicine	C_21_H_24_N_2_O_3_	22.06	353.18652		321.1593	222.1113	210.1121	178.0862	144.0807	[44]
36	3-epi-Ajmalicine or 19-epi-3-iso-Ajmalicine	C_21_H_24_N_2_O_3_	22.34	353.18652		321.1593	222.1112	210.1121	178.0859	144.0806	[44]
37	7-Deoxyloganic acid	C_16_H_24_O_9_	22.35		359.13421	197.0810	153.0907	135.0803	109.0643	89.0228	
38	Kaempferol-*O*-dirhamnosylhexoside	C_33_H_40_O_19_	22.40		739.20856	285.0402	284.0325	283.0244	255.0294	227.0343	
39	Coronaridine	C_21_H_26_N_2_O_2_	22.42	339.20725		307.1795	262.1585	209.1072	144.0807	130.0653	[44]
40	Akuammicine isomer	C_20_H_22_N_2_O_2_	22.75	323.17596		294.1487	291.1490	280.1330	263.1538	234.1289	
41	Strictosidine	C_27_H_34_N_2_O_9_	22.80	531.23426		514.2064	352.1535	334.1432	165.0545	144.0806	[44]
42	Tubotaiwine	C_20_H_24_N_2_O_2_	22.95	325.19161		293.1643	265.1333	236.1421	222.1271	194.0958	[44]
43	Unidentified alkaloid	C_21_H_24_N_2_O_3_	23.06	353.18652		321.1593	228.1015	214.0859	196.0754	168.0805	
44	Unidentified alkaloid	C_20_H_24_N_2_O_2_	23.50	325.19161		296.1642	293.1644	236.1427	216.1016	156.0806	
45	3-epi-Ajmalicine or 19-epi-3-iso-Ajmalicine	C_21_H_24_N_2_O_3_	23.71	353.18652		321.1605	222.1113	210.1121	178.0862	144.0806	[44]
46	Tabersonine or isomer	C_21_H_24_N_2_O_2_	23.76	337.19161		305.1646	277.1695	228.1016	196.0756	168.0806	[42]
47	Serpentine or Alstonine	C_21_H_20_N_2_O_3_	23.80	349.15522		317.1280	263.0811	261.0653	235.0862	206.0829	[45]
48	Serpentine or Alstonine	C_21_H_20_N_2_O_3_	24.44	349.15522		317.1280	263.0810	261.0654	235.0861	206.0832	[45]
49	Tabersonine or isomer	C_21_H_24_N_2_O_2_	24.61	337.19161		305.1642	277.1693	228.1016	196.0758	168.0807	[42]
50	Vindoline	C_25_H_32_N_2_O_6_	24.80	457.23387		439.2197	397.2116	337.1886	222.1125	188.1068	[43,45]
51	Vindolidine	C_24_H_30_N_2_O_5_	25.23	427.22330		409.2113	367.2011	158.0963	143.0730		[43,44]
52	Isorhamnetin-*O*-hexoside	C_22_H_22_O_12_	25.29		477.10330	315.0512	314.0434	285.0407	271.0250	243.0293	
53	Isorhamnetin-3-*O*-rutinoside (Narcissin)	C_28_H_32_O_16_	25.56		623.16122	315.0508	314.0432	300.0276	299.0196	271.0246	
54	Rosicine	C_19_H_20_N_2_O_3_	29.32	325.15522		293.1281	265.1328	249.1381	230.1171	170.0962	[44]
55 ^1^	Isorhamnetin (3′-Methoxy-3,4′,5,7-tetrahydroxyflavone)	C_16_H_12_O_7_	30.41		315.05048	300.0270	151.0026	107.0123			

^1^ Confirmed by standard.

**Table 10:** Chemical composition of callus of *Catharanthus roseus*.

No.	Name	Formula	Rt	[M + H]^+^	[M – H]^−^	Fragment 1	Fragment 2	Fragment 3	Fragment 4	Fragment 5	References
1	Pantothenic acid	C_9_H_17_NO_5_	6.11	220.11850		202.1073	184.0968	174.1122	116.0344	90.0553	
2 ^1^	Tryptamine	C_10_H_12_N_2_	9.65	161.10788		144.0807	143.0730	117.0701	103.0546	91.0547	
3	Unidentified alkaloid	C_20_H_24_N_2_O_2_	13.25	325.19161		307.1801	277.1329	160.1120	152.1068	135.1041	
4	Norharman (β-Carboline)	C_11_H_8_N_2_	14.52	169.07658		115.0542					[44]
5	Loganic acid	C_16_H_24_O_10_	15.70		375.12913	213.0760	169.0857	151.0750	113.0228	69.0329	
6	Vindolinine	C_21_H_24_N_2_O_2_	17.02	337.19161		320.1639	276.1384	177.0908	144.0807	117.0700	[42,43]
7	Secologanoside	C_16_H_22_O_11_	17.25		389.10839	345.1187	209.0444	165.0544	121.0643	69.0329	
8	Sweroside or isomer	C_16_H_22_O_9_	18.00	359.13421		197.0807	179.0702	151.0751	127.0390	111.0806	
9	19-S-Vindolinine	C_21_H_24_N_2_O_2_	18.05	337.19161		320.1640	276.1383	177.0909	144.0807	117.0700	[42,43]
10	Unidentified alkaloid	C_20_H_24_N_2_O_2_	18.08	325.19161		307.1802	277.1330	186.0913	174.0912	138.0914	
11	Unidentified alkaloid	C_25_H_32_N_2_O_6_	18.74	457.23387		439.1856	295.1801	277.1703	185.1084	144.0814	
12	Loganin	C_17_H_26_O_10_	19.02	391.16043		229.1068	197.0818	179.0703	151.0752	109.0651	[45]
13	Unidentified alkaloid	C_25_H_32_N_2_O_6_	19.36	457.23387		325.1898	307.1802	270.1330	174.0914	122.0963	
14	Unidentified alkaloid	C_20_H_22_N_2_O_2_	19.60	323.17596		216.1016	184.0755	156.0806	129.0700		
15	Harmine isomer	C_13_H_12_N_2_O	19.63	213.10279		198.0786	170.0833	88.0760			
16	Vinervine	C_20_H_22_N_2_O_3_	20.02	339.17087		307.1436	279.1490	250.1216	185.0705		
17	Panarine	C_20_H_22_N_2_O_2_	20.25	323.17596		305.1641	166.0861	156.0805	148.1120	144.0807	
18	Secologanol	C_17_H_26_O_10_	20.34	391.16043		229.1065	211.0961	193.0858	179.0700	167.0700	
19	Unidentified alkaloid	C_21_H_24_N_2_O_2_	20.35	337.19161		305.1643	277.1690	234.1276	196.0995	144.0805	
20	Antirhine	C_19_H_24_N_2_O	20.83	297.19669		280.1689	196.1122	166.1227	154.1225	144.0807	[44]
21	Unidentified alkaloid	C_21_H_24_N_2_O_2_	20.93	337.19161		305.1647	277.1700	222.1274	180.1019	156.0807	
22	Cathenamine or Vallesiachotamine	C_21_H_22_N_2_O_3_	21.22	351.17087		321.1590	289.1335	247.1226	233.1069	182.0838	[44]
23	11-Hydroxycyclolochnerine or Lochneridine	C_20_H_24_N_2_O_3_	21.46	341.18652		323.1748	279.1488	264.1354	198.0911		[44]
24	Cathenamine or Vallesiachotamine	C_21_H_22_N_2_O_3_	21.56	351.17087		321.1593	289.1331	247.1226	168.0804		[44]
25	Akuammicine	C_20_H_22_N_2_O_2_	21.65	323.17596		294.1487	291.1487	280.1311	263.1538	234.1280	[44]
26	Catharanthine	C_21_H_24_N_2_O_2_	21.82	337.19161		173.1071	165.0906	144.0806	133.0648	93.0702	[43,45]
27	Ajmalicine	C_21_H_24_N_2_O_3_	22.01	353.18652		321.1586	222.1112	210.1121	178.0860	144.0807	[44]
28	7-Deoxyloganic acid	C_16_H_24_O_9_	22.33		359.13421	197.0811	153.0907	135.0801	109.0644	89.0227	
29	3-epi-Ajmalicine or 19-epi-3-iso-Ajmalicine	C_21_H_24_N_2_O_3_	22.36	353.18652		321.1593	222.1112	210.1122	178.0860	144.0806	[44]
30	Strictosidine	C_27_H_34_N_2_O_9_	22.63	531.23426		514.2069	352.1545	334.1433	165.0544	144.0807	[44]
31	Tubotaiwine	C_20_H_24_N_2_O_2_	22.83	325.19161		293.1643	265.1325	236.1427	222.1264	194.0966	[44]
32	Tabersonine or isomer	C_21_H_24_N_2_O_2_	23.68	337.19161		305.1645	277.1696	228.1014	196.0759	168.0805	[42]
33	Serpentine or Alstonine	C_21_H_20_N_2_O_3_	23.70	349.15522		317.1278	263.0810	261.0652	235.0862	206.0832	[45]
34	Serpentine or Alstonine	C_21_H_20_N_2_O_3_	24.40	349.15522		317.1279	263.0810	261.0653	235.0860	206.0827	[45]
35	Vindoline	C_25_H_32_N_2_O_6_	24.84	457.23387		439.2195	397.2118	337.1883	222.1122	188.1069	[43,45]
36	Unidentified alkaloid	C_21_H_24_N_2_O_3_	24.93	353.18652		321.1592	293.1629	250.1233	212.0932	199.0865	
37	Vindolidine	C_24_H_30_N_2_O_5_	25.37	427.22330		409.2098	367.2010	158.0962	143.0727		[43,44]
38	Unidentified alkaloid	C_21_H_24_N_2_O_3_	26.34	353.18652		321.1595	278.1180	210.1122	170.0959	144.0807	
39	Rosicine	C_19_H_20_N_2_O_3_	29.33	325.15522		293.1280	265.1329	249.1381	230.1171	170.0962	[44]

^1^ Confirmed by standard.

**Table 11:** Chemical composition of normal green shoot tissues of *Catharanthus roseus*.

No.	Name	Formula	Rt	[M + H]^+^	[M – H]^−^	Fragment 1	Fragment 2	Fragment 3	Fragment 4	Fragment 5	References
1	Neochlorogenic acid (5-*O*-Caffeoylquinic acid)	C_16_H_18_O_9_	10.12	355.10291		163.0387	145.0283	135.0440	117.0336	89.0388	
2	Unidentified alkaloid	C_20_H_24_N_2_O_2_	13.27	325.19161		307.1799	277.1329	160.1117	152.1068	135.1042	
3	3-*O*-Feruloylquinic acid cis isomer	C_17_H_20_O_9_	14.84		367.10291	193.0498	191.0550	173.0444	134.0360		
4 ^1^	Chlorogenic acid (3-*O*-Caffeoylquinic acid)	C_16_H_18_O_9_	14.87	355.10291		163.0387	145.0283	135.0440	117.0337	89.0389	
5	3-*O*-Feruloylquinic acid	C_17_H_20_O_9_	15.09		367.10291	193.0497	191.0552	173,0443	134.0360	93.0329	
6	Loganic acid	C_16_H_24_O_10_	15.70		375.12913	213.0760	169.0858	151.0751	113.0229	69.0329	
7	Chryptochlorogenic acid (4-*O*-Caffeoylquinic acid)	C_16_H_18_O_9_	16.11	355.10291		163.0388	145.0284	135.0441	117.0336	89.0389	
8	Vindolinine	C_21_H_24_N_2_O_2_	17.02	337.19161		320.1640	276.1380	177.0910	144.0807	117.0700	[42,43]
9	Secologanoside	C_16_H_22_O_11_	17.25		389.10839	345.1189	209.0446	165.0543	121.0643	69.0329	
10	5-*O*-(4-Coumaroyl)quinic acid	C_16_H_18_O_8_	17.40		337.09235	191.0552	173.0443	163.0388	119.0487	93.0329	
11	4-*O*-Feruloylquinic acid cis isomer	C_17_H_20_O_9_	17.59		367.10291	193.0496	191.0556	173.0443	134.0360	93.0329	
12	Sweroside or isomer	C_16_H_22_O_9_	17.98	359.13421		197.0807	179.0701	151.0752	127.0390	111.0806	
13	Unidentified alkaloid	C_20_H_24_N_2_O_2_	17.99	325.19161		307.1800	277.1325	186.0914	174.0912	138.0913	
14	4-*O*-(4-Coumaroyl)quinic acid	C_16_H_18_O_8_	18.04		337.09235	191.0550	173.0443	163.0387	119.0486	93.0329	
15	19-*S*-Vindolinine	C_21_H_24_N_2_O_2_	18.07	337.19161		320.1640	276.1385	177.0908	144.0807	117.0700	[42,43]
16	Unidentified alkaloid	C_20_H_22_N_2_O_2_	18.10	323.17596		248.1431	219.1040	173.1070	144.0806	79.0547	
17	5-*O*-Feruloylquinic acid	C_17_H_20_O_9_	18.45		367.10291	193.0499	191.0552	173.0443	134.0359	93.0329	
18	Unidentified alkaloid	C_21_H_28_N_2_O_3_	18.88	357.21782		253.1695	226.1434	214.1434	144.0806	110.0966	
19	4-*O*-Feruloylquinic acid	C_17_H_20_O_9_	18.95		367.10291	193.0497	191.0548	173.0443	134.0360	93.0329	
20	Unidentified alkaloid	C_20_H_22_N_2_O_2_	19.59	323.17596		216.1016	184.0757	156.0806	129.0702		
21	5-*O*-(4-Coumaroyl)quinic acid cis isomer	C_16_H_18_O_8_	19,63		337.09235	191.0552	173.0440	163.0391	119.0487	93.0328	
22	Antirhine isomer	C_19_H_24_N_2_O	19.77	297.19669		280.1697	236.1425	166.1225	154.1224	144.0807	
23	11-Hydroxycyclolochnerine or Lochneridine	C_20_H_24_N_2_O_3_	19.84	341.18652		323.1751	281.1640	264.1386	218.0808	200.0703	[44]
24	Vinervine	C_20_H_22_N_2_O_3_	19.98	339.17087		307.1436	279.1487	250.1258	185.0707		
25	Methyl caffeoylquinate	C_17_H_20_O_9_	19.99		367.10291	193.0499	179.0340	173.0443	161.0232	135.0438	
26	Panarine	C_20_H_22_N_2_O_2_	20.26	323.17596		305.1644	166.0860	156.0805	148.1119	144.0807	
27	Secologanol	C_17_H_26_O_10_	20.33	391.16043		229.1069	211.0963	193.0859	179.0702	167.0703	
28	5-*O*-Feruloylquinic acid cis isomer	C_17_H_20_O_9_	20.48		367.10291	193.0499	191.0552	173.0448	134.0361	93.0329	
29	Ammocalline	C_19_H_22_N_2_	20.54	279.18612		248.1429	219.1041	149.0231	144.0806	107.0858	[44]
30	Antirhine	C_19_H_24_N_2_O	20.75	297.19669		280.1687	196.1117	166.1225	154.1225	144.0807	[44]
31	Unidentified alkaloid	C_21_H_24_N_2_O_2_	20.81	337.19161		305.1640	222.1275	180.1017	156.0807	144.0806	
32	Quercetin-*O*-dirhamnosylhexoside	C_33_H_40_O_20_	21.02		755.20347	301.0352	300.0275	299.0216	271.0247	255.0294	
33	Cathenamine or Vallesiachotamine	C_21_H_22_N_2_O_3_	21.17	351.17087		321.1596	289.1333	247.1226	233.1069	182.0837	[44]
34	11-Hydroxycyclolochnerine or Lochneridine	C_20_H_24_N_2_O_3_	21.38	341.18652		323.1749	279.1491	264.1279	198.0913	138.1277	[44]
35	Cathenamine or Vallesiachotamine	C_21_H_22_N_2_O_3_	21.42	351.17087		321.1592	289.1331	247.1223	196.0756	168.0806	[44]
36	Akuammicine	C_20_H_22_N_2_O_2_	21.49	323.17596		294.1485	291.1487	280.1332	263.1538	234.1279	[44]
37	Catharanthine	C_21_H_24_N_2_O_2_	21.57	337.19161		173.1071	165.0908	144.0806	133.0648	93.0702	[43,45]
38	Desacetylvindoline	C_23_H_30_N_2_O_5_	21.91	415.22330		397.2130	365.1854	355.2009	188.1069	173.0830	
39	Ajmalicine	C_21_H_24_N_2_O_3_	21.99	353.18652		321.1596	222.1119	210.1122	178.0863	144.0807	[44]
40	Coronaridine	C_21_H_26_N_2_O_2_	22.29	339.20725		307.1802	262.1590	209.1062	144.0808	130.0646	[44]
41	7-Deoxyloganic acid	C_16_H_24_O_9_	22.33		359.13421	197.0811	153.0907	135.0802	109.0643	89.0228	
42	Kaempferol-*O*-dirhamnosylhexoside	C_33_H_40_O_19_	22.38		739.20856	285.0403	284.0326	283.0246	255.0295	227.0341	
43	Akuammicine isomer	C_20_H_22_N_2_O_2_	22.70	323.17596		294.1486	291.1487	280.1325	263.1538	234.1281	
44	Strictosidine	C_27_H_34_N_2_O_9_	22.77	531.23426		514.2066	352.1537	334.1431	165.0543	144.0807	[44]
45	Tubotaiwine	C_20_H_24_N_2_O_2_	22.89	325.19161		293.1642	265.1330	236.1440	222.1274	194.0963	[44]
46	Unidentified alkaloid	C_21_H_24_N_2_O_3_	23.02	353.18652		321.1590	228.1014	214.0865	196.0755	168.0805	
47	Unidentified alkaloid	C_20_H_24_N_2_O_2_	23.45	325.19161		296.1636	293.1644	236.1434	216.1016	156.0806	
48	Serpentine or Alstonine	C_21_H_20_N_2_O_3_	23.71	349.15522		317.1280	263.0810	261.0653	235.0863	206.0832	[45]
49	Tabersonine or isomer	C_21_H_24_N_2_O_2_	23.72	337.19161		305.1646	277.1698	228.1016	196.0755	168.0806	[42]
50	Unidentified alkaloid	C_21_H_24_N_2_O_3_	24.13	353.18652		336.1828	308.1645	229.1096	165.0908	144.0807	
51	Serpentine or Alstonine	C_21_H_20_N_2_O_3_	24.32	349.15522		317.1278	263.0810	261.0654	235.0862	206.0825	[45]
52	Tabersonine or isomer	C_21_H_24_N_2_O_2_	24.54	337.19161		305.1643	277.1692	228.1017	196.0753	168.0807	[42]
53	Vindoline	C_25_H_32_N_2_O_6_	24.65	457.23387		439.2211	397.2116	337.1901	222.1122	188.1068	[43,45]
54	Vindolidine	C_24_H_30_N_2_O_5_	24.96	427.22330		409.2123	367.2012	158.0963	143.0727		[43,44]
55	Isorhamnetin-O-hexoside	C_22_H_22_O_12_	25.28		477.10330	315.0509	314.0433	285.0404	271.0247	243.0294	
56	Isorhamnetin-3-O-rutinoside (Narcissin)	C_28_H_32_O_16_	25.56		623.16122	315.0511	314.0433	300.0275	299.0197	271.0249	
57	Methoxy-trihydroxyflavanone	C_16_H_14_O_6_	27.83	303.08686		179.0337	177.0546	163.0394	153.0181	145.0284	
58	Rosicine	C_19_H_20_N_2_O_3_	29.33	325.15522		293.1284	265.1332	249.1384	230.1173	170.0962	[44]
59 ^1^	Isorhamnetin (3′-Methoxy-3,4′,5,7-tetrahydroxyflavone)	C_16_H_12_O_7_	30.40		315.05048	300.0270	283.0252	271.0257	151.0022	107.0122	

^1^ Confirmed by standard.

**Table 12:** Antioxidant parameters of the tested extracts (IC50 (mg/mL)).

	DPPH	ABTS	CUPRAC	FRAP	PBD	Chelating
Somaclonal variant shoot	1.65 ± 0.05	1.45 ± 0.01	1.33 ± 0.01	1.00 ± 0.01	1.44 ± 0.04	0.69 ± 0.02
Callus	>3	1.85 ± 0.05	2.41 ± 0.01	1.35 ± 0.02	>3	0.96 ± 0.02
Normal green shoot	1.57 ± 0.08	1.44 ± 0.03	1.16 ± 0.01	0.97 ± 0.01	1.13 ± 0.06	0.82 ± 0.02
Trolox	0.06 ± 0.01	0.09 ± 0.01	0.11 ± 0.01	0.04 ± 0.01	0.52 ± 0.02	nt
EDTA	nt	nt	nt	nt	nt	0.02 ± 0.001

Values are expressed as mean ± S.D. of three parallel measurements. nt, not tested; ethylenediaminetetraacetic acid: EDTA; DPPH: 2,2-diphenyl-1-picrylhydrazyl; ABTS: 2,2′-azino-bis(3-ethylbenzothiazoline-6-sulphonic acid); CUPRAC: Cupric reducing antioxidant capacity; FRAP: Ferric reducing antioxidant power; PBD, phosphomolybdenum.

**Table 13:** Enzyme inhibitory effects of the tested extracts (IC50 (mg/mL)).

	AChE	BChE	Tyrosinase	Amylase
Somaclonal variant shoot	0.74 ± 0.01	1.02 ± 0.03	0.86 ± 0.02	1.39 ± 0.02
Callus	0.65 ± 0.01	1.04 ± 0.03	1.05 ± 0.03	1.29 ± 0.07
Normal green shoot	0.72 ± 0.01	0.96 ± 0.06	0.83 ± 0.01	1.30 ± 0.01
Galantamine	0.003 ± 0.001	0.007 ± 0.002	nt	nt
Kojic acid	nt	Nt	0.08 ± 0.001	nt
Acarbose	nt	Nt	nt	0.68 ± 0.01

Values are expressed as mean ± S.D. of three parallel measurements. nt, not tested.

## Supplementary Materials

Supplementary Materials are available online. Figure S1: Total ion chromatogram of the albino shoot sample in positive mode; Figure S2: Total ion chromatogram of the albino shoot sample in positive mode in 13–28 min; Figure S3: Total ion chromatogram of albino shoot sample in negative mode; Figure S4: Total ion chromatogram of callus sample in positive mode; Figure S5: Total ion chromatogram of callus sample in positive mode in 11–28 min; Figure S6: Total ion chromatogram of callus sample in negative mode; Figure S7: Total ion chromatogram of the normal-green shoot sample in positive mode; Figure S8: Total ion chromatogram of the normal-green shoot sample in a positive mode in 14–28 min; Figure S9: Total ion chromatogram of the normal-green shoot sample in negative mode; Figure S10: The typical extracted ion chromatogram (*m*/*z* 337.1916) in positive ion mode; Figure S11: MS2 spectrum of Catharantine at retention time 21.76 min; Figure S12: The typical extracted ion chromatogram (*m*/*z* 427.2233) in positive ion mode; Figure S13: MS2 spectrum of Vindolidine at retention time 25.23 min
