# Supplementary material for: Phytochemical Composition, Antioxidant Capacity, and Enzyme Inhibitory Activity in Callus, Somaclonal Variant, and Normal Green Shoot Tissues of Catharanthus roseus (L) G. Don
Source: Molecules. 2020 Oct 26;25(21):4945. doi: 10.3390/molecules25214945 (PMC7663286; doi:10.3390/molecules25214945)
Supplement: Supplementary file 1 [file molecules-25-04945-s001.pdf]

Phytochemical composition, antioxidant capacity, and enzyme inhibitory activity in callus, somaclonal variant, and normal-green shoot tissues of *Catharanthus roseus* (L) G. Don

Chemical composition

Chemical compositions of the *Catharanthus roseus* extracts were determined using a Dionex Ultimate 3000RS UHPLC instrument. The extracts were filtered through 0.22 μm PTFE syringe filter (Labex Ltd, Hungary) before HPLC analysis. The compounds were separated on a Thermo Accucore C18 (100 mm x 2.1, mm i. d., 2.6 μm) column thermostated at 25 °C (± 1 °C). The solvents used were water (A) and methanol (B); both were acidified with 0.1% formic acid. The flow rate was maintained at 0.2 mL min^{-1}. The elution gradient was isocratic 5% B (0-3 min), a linear gradient increasing from 5% B to 100% (3-43 min), 100% B (43-61 min), a linear gradient decreasing from 100% B to 5% (61-62 min) and 5% B (62-70 min). The column was coupled to a Thermo Q Exactive Orbitrap mass spectrometer (Thermo Scientific, USA) equipped with an electrospray ionization source. MS spectra were recorded in positive and negative-ion mode, respectively.

Trace Finder 3.1 (Thermo Scientific, USA) software was applied for target screening. The compounds listed in the tables were identified on the basis of our previously published works or data found in the literature using exact molecular mass, isotopic pattern, and characteristic fragment ions. In every case, the exact molecular mass, isotopic pattern, characteristic fragment ions, and retention time (min) were used for the identification of the compounds which were confirmed by standards.

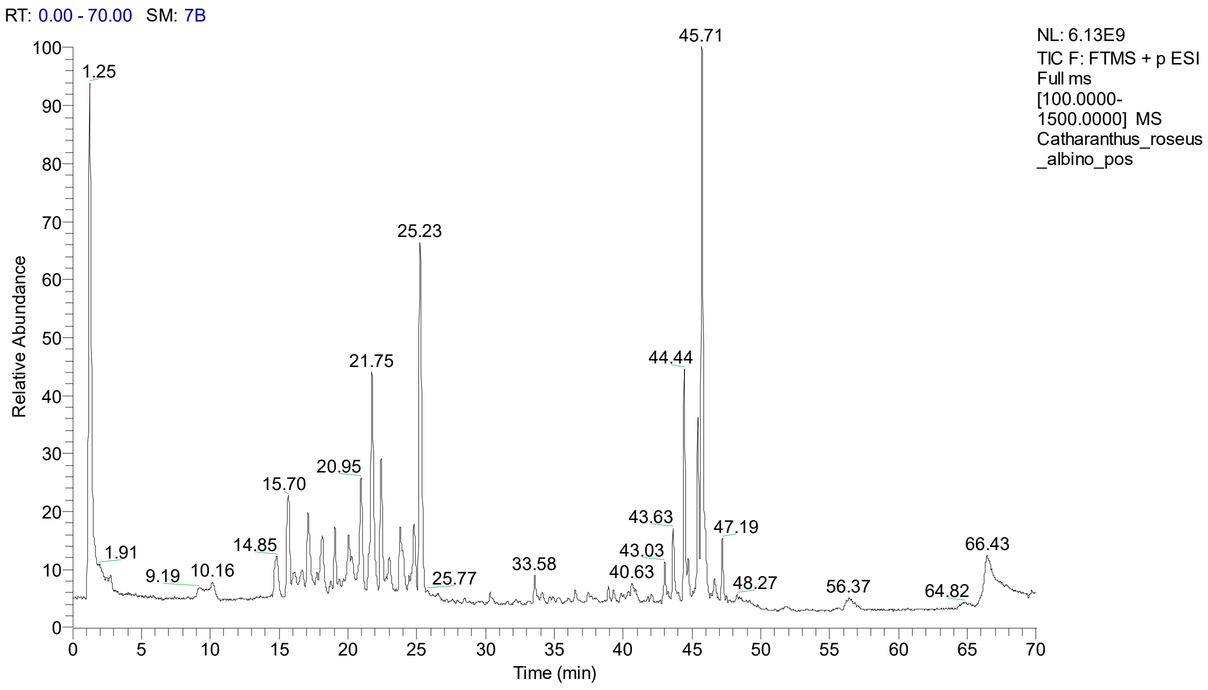

Figure S1. Total ion chromatogram of the albino shoot sample in positive mode

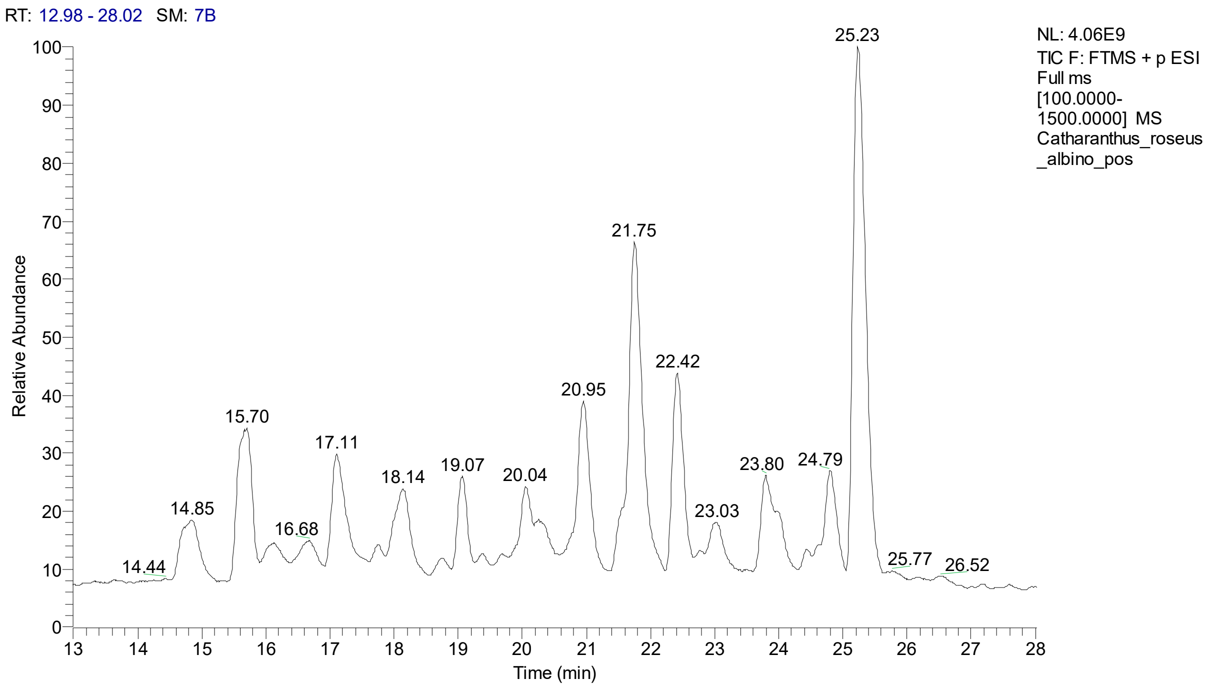

Figure S2. Total ion chromatogram of the albino shoot sample in positive mode in 13-28 min

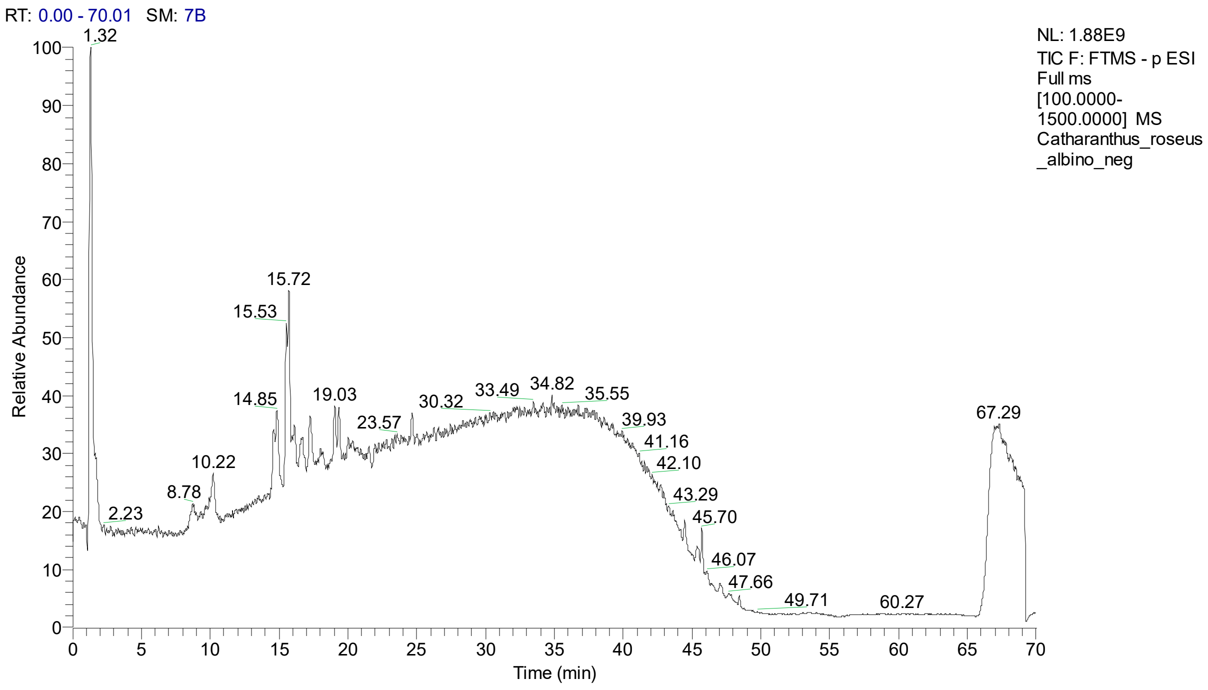

Figure S3. Total ion chromatogram of albino shoot sample in negative mode

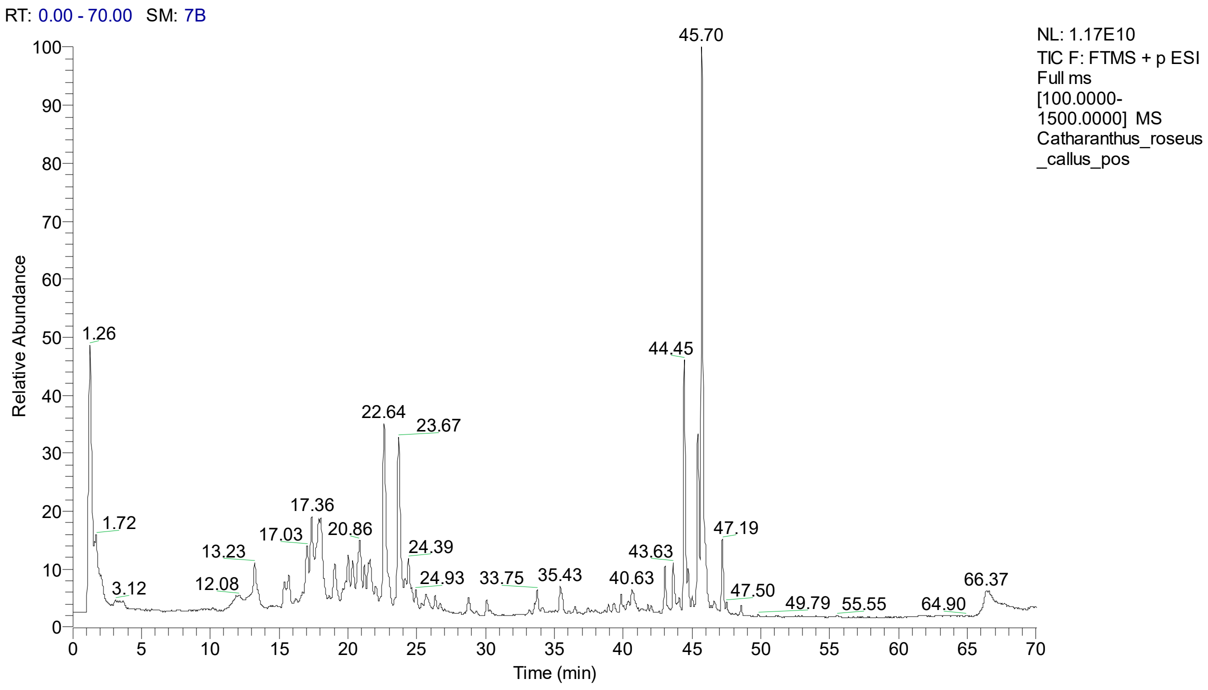

Figure S4. Total ion chromatogram of callus sample in positive mode

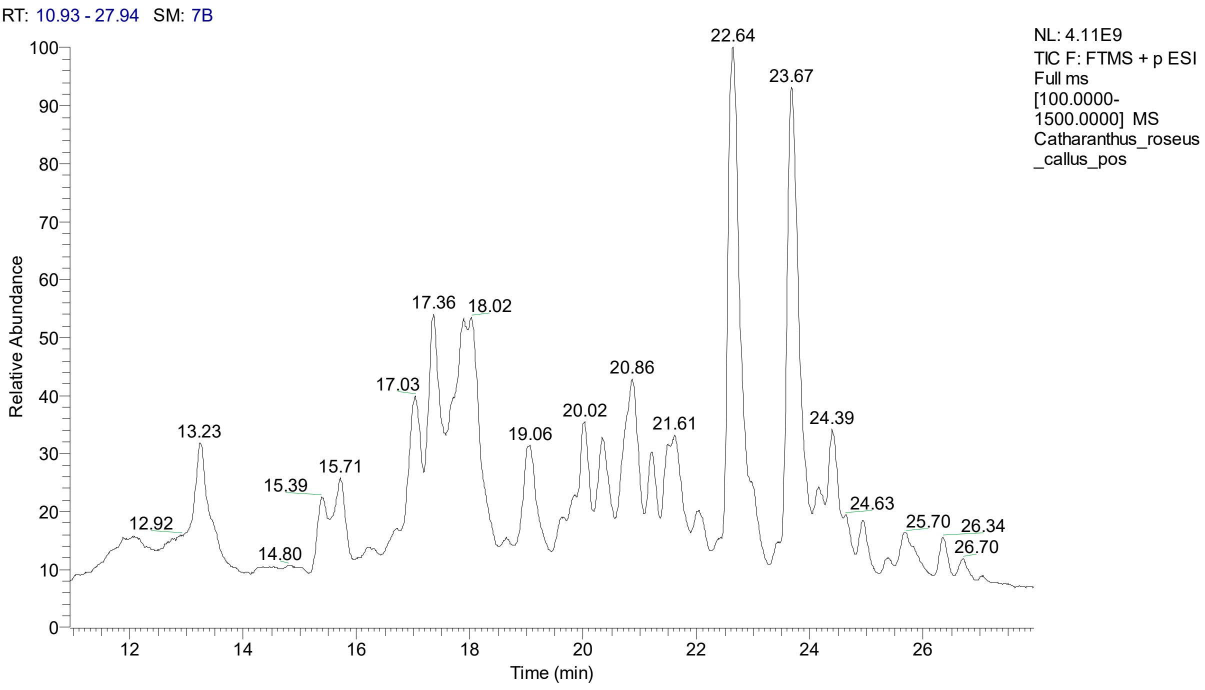

Figure S5. Total ion chromatogram of callus sample in positive mode in 11-28 min.

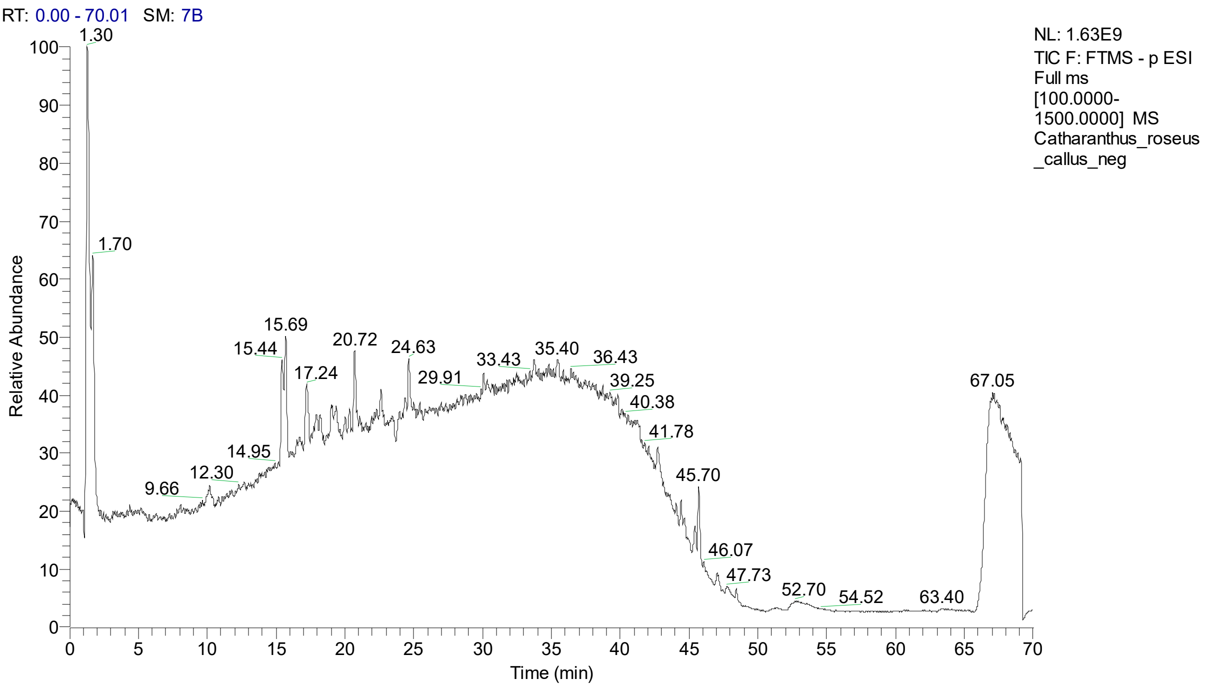

Figure S6. Total ion chromatogram of callus sample in negative mode

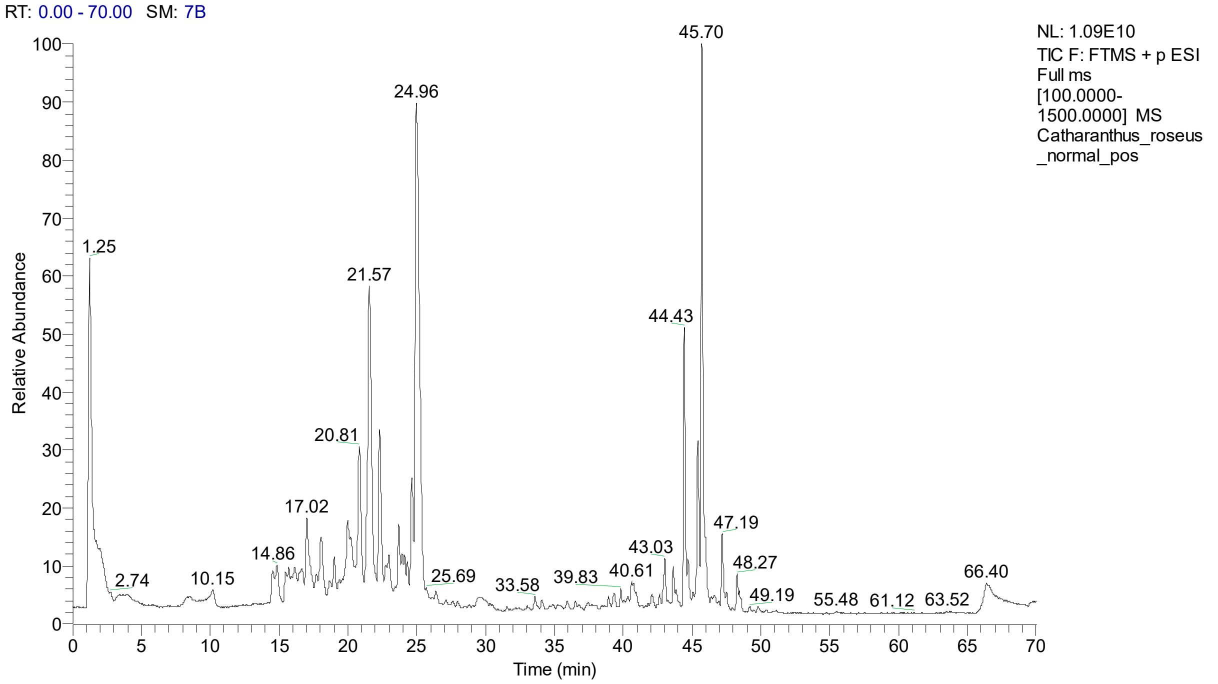

Figure S7. Total ion chromatogram of the normal-green shoot sample in positive mode

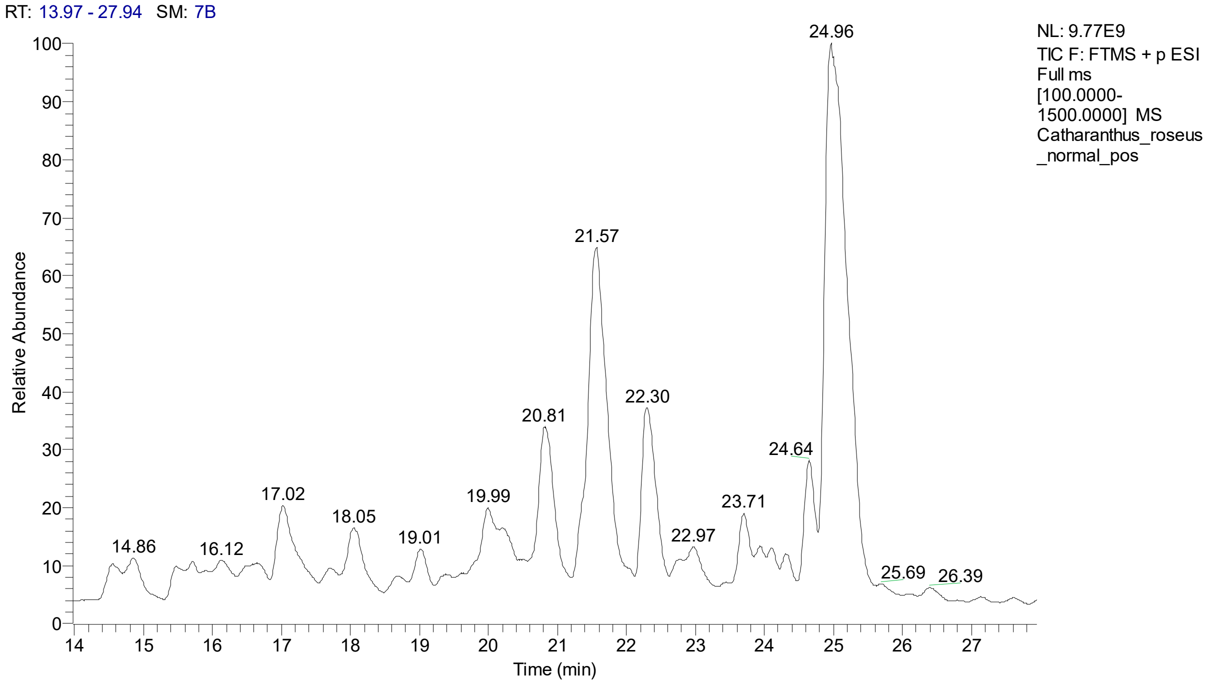

Figure S8. Total ion chromatogram of the normal-green shoot sample in a positive mode in 14-28 min

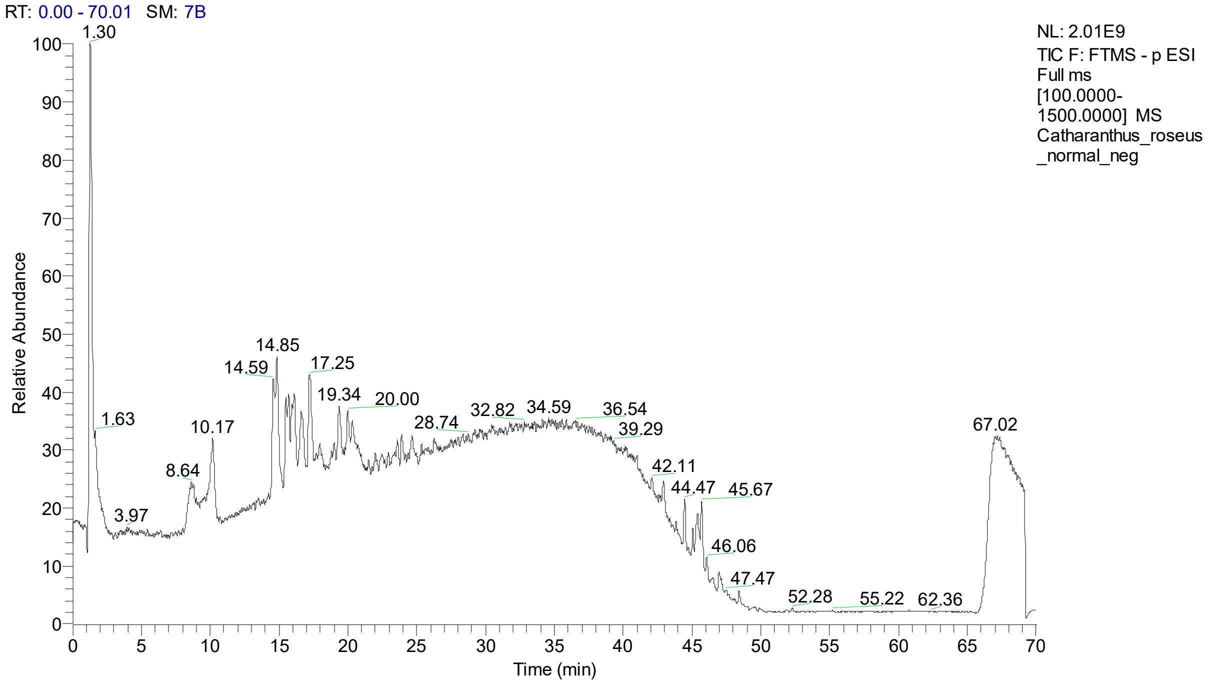

Figure S9. Total ion chromatogram of the normal-green shoot sample in negative mode

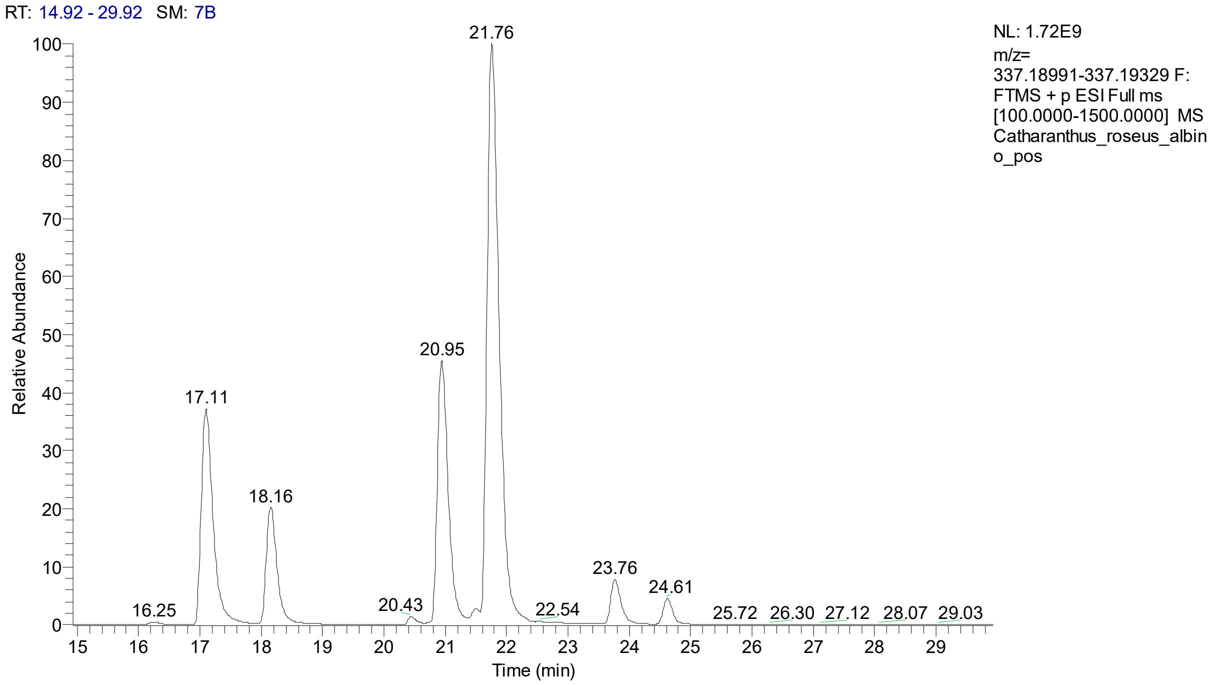

Figure S10. The typical extracted ion chromatogram (m/z 337.1916) in positive ion mode

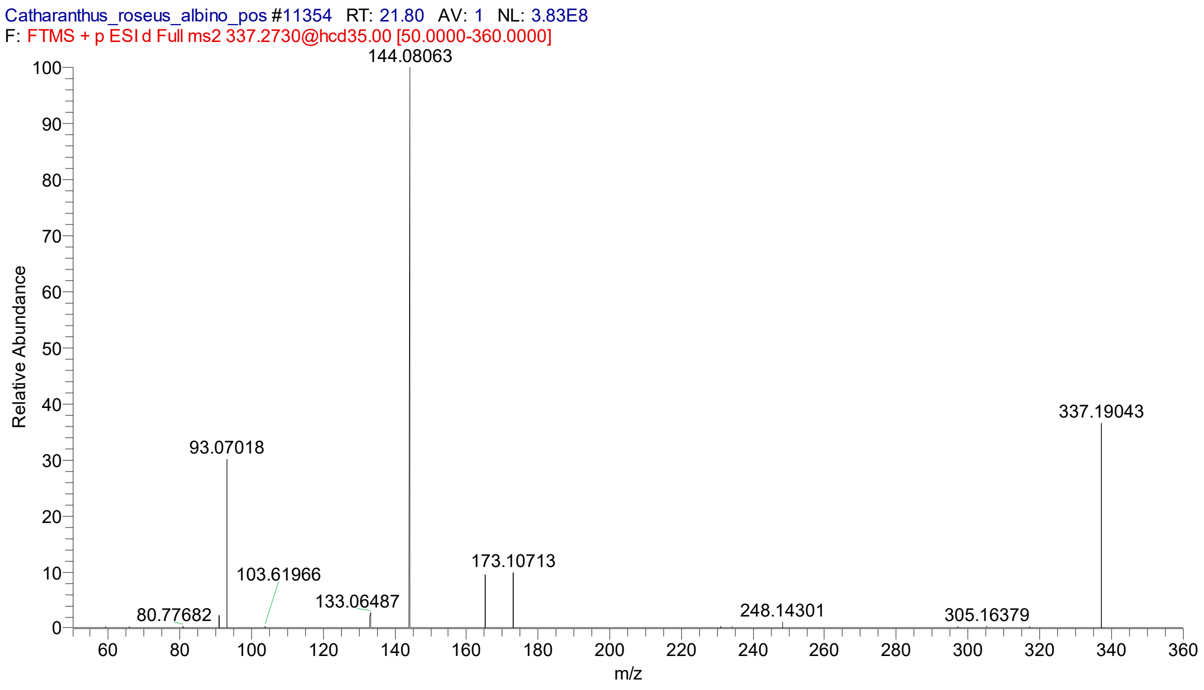

Figure S11. MS2 spectrum of Catharantine at retention time 21.76 minutes

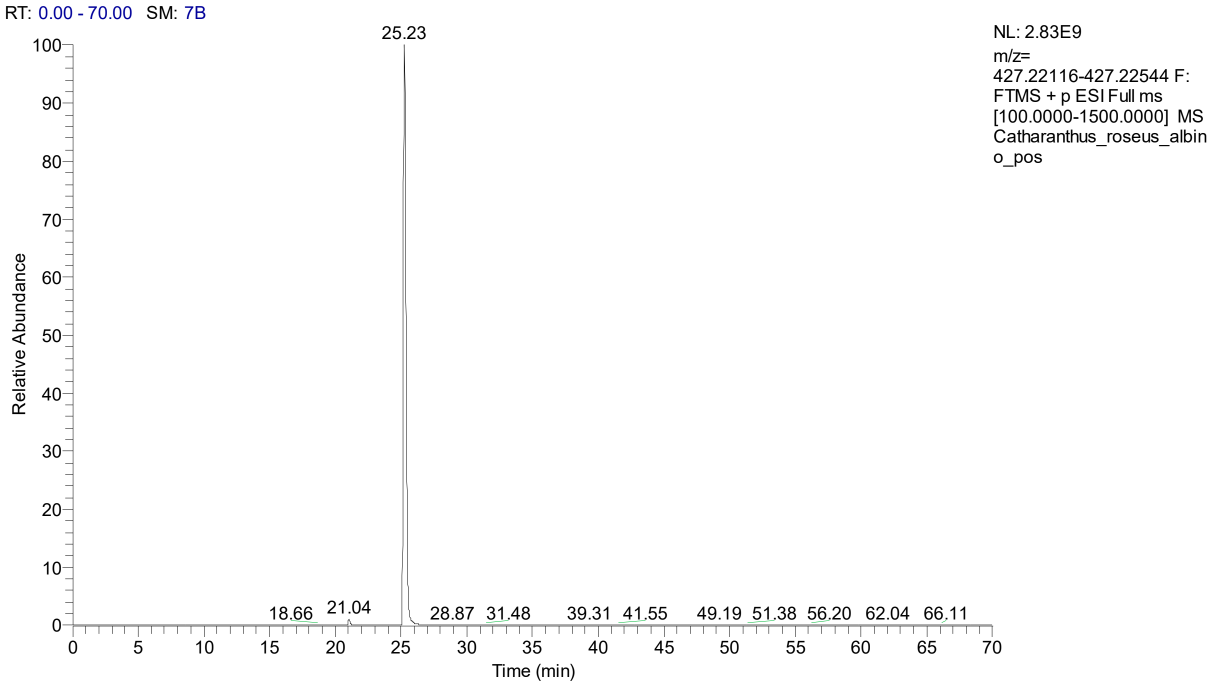

Figure S12. The typical extracted ion chromatogram (m/z 427.2233) in positive ion mode

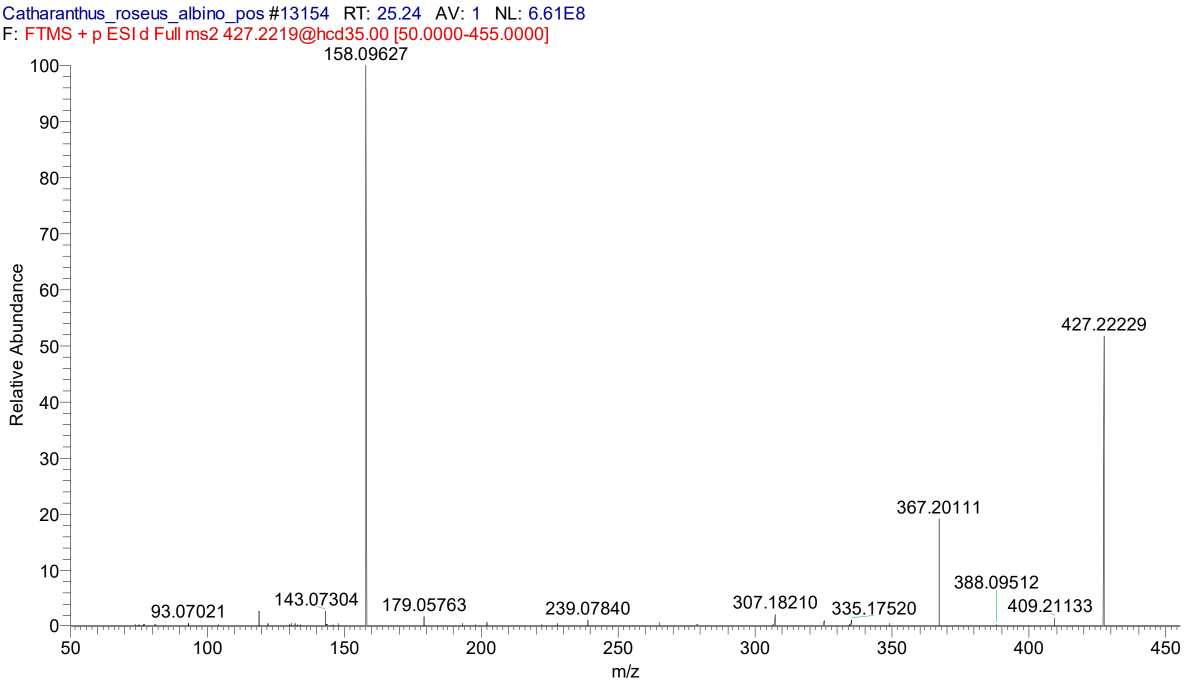

Figure S13. MS2 spectrum of Vindolidine at retention time 25.23 minutes
